# *Listeria monocytogenes* GshF contributes to oxidative stress tolerance via regulation of the phosphoenolpyruvate-carbohydrate phosphotransferase system

**DOI:** 10.1128/spectrum.02365-23

**Published:** 2023-09-05

**Authors:** Mianmian Chen, Jiaxue Zhang, Jing Xia, Jing Sun, Xian Zhang, Jiali Xu, Simin Deng, Yue Han, Lingli Jiang, Houhui Song, Changyong Cheng

**Affiliations:** 1 Key Laboratory of Applied Technology on Green-Eco-Healthy Animal Husbandry of Zhejiang Province, College of Animal Science and Technology & College of Veterinary Medicine, Zhejiang A&F University, Hangzhou, China; 2 Zhejiang Provincial Engineering Research Center for Animal Health Diagnostics & Advanced Technology, College of Animal Science and Technology & College of Veterinary Medicine, Zhejiang A&F University, Hangzhou, China; 3 Zhejiang International Science and Technology Cooperation Base for Veterinary Medicine and Health Management, College of Animal Science and Technology & College of Veterinary Medicine, Zhejiang A&F University, Hangzhou, China; 4 China-Australia Joint Laboratory for Animal Health Big Data Analytics, College of Animal Science and Technology & College of Veterinary Medicine, Zhejiang A&F University, Hangzhou, China; 5 Ningbo College of Health Sciences, Ningbo, China; Yangzhou University, Yangzhou, Jiangsu, China

**Keywords:** *Listeria monocytogenes*, glutathione synthase GshF, oxidative tolerance, *iiB^man^*

## Abstract

Glutathione (GSH) is an essential component of the glutaredoxin (Grx) system, and it is synthesized by the enzyme glutathione synthase GshF in *Listeria monocytogenes*. GSH plays a crucial role in regulating *Listeria* virulence by modifying the virulence factors LLO and PrfA. In this study, we investigated the involvement of *L. monocytogenes* GshF in oxidative tolerance and intracellular infection. Our findings revealed that the deletion of *gshF* resulted in a significant reduction in bacterial growth *in vitro* when exposed to diamide and copper ions stress. More importantly, this deletion also impaired the efficiency of invasion and proliferation in macrophages and mice organs. Furthermore, GshF influenced global transcriptional profiles, including carbohydrate and amino acid metabolism, particularly those related to the phosphoenolpyruvate-carbohydrate phosphotransferase system (PTS) genes *lmo1997-lmo2004*, under oxidative stress conditions. In the wild-type strain, the transcription of *lmo1997-lmo2004* was notably downregulated in response to copper ions and diamide stress compared to normal conditions. However, in the absence of *gshF*, the transcripts of *lmo1997-lmo2004* were upregulated in response to these stress conditions. Notably, the deletion of *iiB^man^* (*lmo2002*) enhanced oxidative stress tolerance to copper ions, whereas overexpression of *iiB^man^* reduced this resistance. In conclusion, our study provides the first evidence that *L. monocytogenes* GshF plays a crucial role in bacterial antioxidation through the regulation of *iiB^man^*.

**IMPORTANCE:**

*Listeria monocytogenes* has developed various mechanisms to withstand oxidative stress, including the thioredoxin and glutaredoxin systems. However, the specific role of the glutathione synthase GshF, responsible for synthesizing GSH in *L. monocytogenes*, in oxidative tolerance remains unclear. This study aimed to elucidate the relationship between GshF and oxidative tolerance in *L. monocytogenes* by examining the efficiency of invasion and proliferation in macrophages and mice organs, as well as analyzing global transcriptional profiles under oxidative stress conditions. The results revealed that GshF plays a significant role in *L. monocytogenes*’ response to oxidative stress. Notably, GshF acts to suppress the transcription of phosphoenolpyruvate-carbohydrate phosphotransferase system genes *lmo1997-lmo2004*, among which *iiB^man^* (*lmo2002*) was identified as the most critical gene for resisting oxidative stress. These findings enhance our understanding of how *L. monocytogenes* adapts to its environment and provide valuable insights for investigating the environmental adaptation mechanisms of other pathogenic bacteria.

## INTRODUCTION

*Listeria monocytogenes* is a gram-positive facultative intracellular pathogen that causes listeriosis, a systemic disease (1). It poses a significant health risk, particularly to immunocompromised individuals and pregnant women, with high mortality rates among foodborne pathogens (2, 3). *L. monocytogenes* is commonly found in food processing environments (4, 5) and can be detected in various food sources, including fresh milk, vegetables, and fruit (6, 7). The primary route of *L. monocytogenes* infection is through the consumption of contaminated food (8).

Bacteria encounter oxidative stresses in their natural environment and during host infection, including reactive oxygen species (ROS), reactive nitrogen species (RNS), and reactive chlorine species (RCS), generated in hostile bacterial environments such as macrophages (9). These stresses can damage cellular components, including DNA, membrane lipids, and proteins (9, 10). To counteract oxidative stress, *L. monocytogenes* has evolved mechanisms such as the thioredoxin (Trx) and glutaredoxin (Grx) systems. These systems facilitate thiol-disulfide exchange reactions, redox sensing, regulation of protein thiol function, cellular redox homeostasis, and oxidative protein folding (9, 11, 12). The glutaredoxin system consists of NADPH, glutathione reductase (GR), GSH, and Grx (9). In oxidative stress conditions, oxidized Grxs react with target disulfides, becoming reduced when the disulfide is reduced, and subsequently react with two GSH molecules to regenerate reduced (9). GSH acts as a redox buffer, protecting cells from oxidative damage by reacting with ROS, RCS, and RNS. It also forms reversible glutathione-protein mixed disulfides with protein cysteine residues, a reversible post-translational modification known as Cys-S-glutathionylation, which prevents irreversible oxidation of cysteine residues and regulates protein activity (9, 13).

GSH has been found to regulate bacterial pathogenesis. For example, *Pseudomonas aeruginosa* lacking GSH biosynthesis genes exhibits attenuation in a mouse model (14, 15). GSH in *P. aeruginosa* functions as an intracellular redox signal sensed by Vfr, leading to upregulated expression of the type III secretion system (T3SS) (16). In *Streptococcus pyogenes*, GSH increases the intracellular threshold of copper ions tolerance (17) and is utilized by the bacteria for growth and evasion of the host’s innate immune response (18). In *L. monocytogenes*, GSH synthesized by GshF (encoded by *lmo2770*) is a component of the Grx system and plays a crucial role in maintaining intracellular redox homeostasis (19). Importantly, GSH has been shown to regulate the virulence activity of PrfA and LLO proteins through allosteric activation and reversible post-translational modification (S-glutathionylation), respectively (20 – 22). However, how *L. monocytogenes* GshF is able to resist oxidative stress has not been investigated, prompting us to elucidate the molecular functions and underlying molecular basis of GshF during bacterial environmental adaptation and intracellular infection.

## MATERIALS AND METHODS

### Bacterial strains, plasmids, primers, and growth conditions

The recombinant *L. monocytogenes* strains used were all on the wild-type EGD-e background. *Escherichia coli* DH5α was used for cloning experiments and as the host strain for plasmids pAM401, pIMK2, and pKSV7. *L. monocytogenes* and *E.coli* were routinely cultured in Brain Heart Infusion (BHI, Oxoid) and Lysogeny Broth (LB, Oxoid), respectively, at 37°C aerobically with shaking. BHI agar and LB agar plates were used for growth on solid media. Antibiotics were added to the media as appropriate: ampicillin 50 mg/mL, kanamycin 50 mg/mL, or chloramphenicol 10 mg/mL. All primers are listed in Table S1.

### In-frame deletion and complementation of *L. monocytogenes* genes

To prepare electrocompetent *L. monocytogenes*, first, the bacteria to mid-log phase [approximately optical density (OD)_600 nm_ 0.18–0.22, ensuring it does not exceed OD_600 nm_ 0.25] were grown. Next, penicillin G was added to a final concentration of 20 µg/mL and allowed to continue its growth for 2 hours. Afterward, the cells were pelleted, washed, and resuspended in HEPES-Sucrose solution. For the gene deletion using the homologous recombination strategy, the temperature-sensitive pKSV7 shuttle plasmid was utilized. Briefly, the electroporation parameters of the electroporator (Bio-Rad) were set to 210 ohms, 2 kV, and 25 µF, and then the recombinant plasmid containing the upstream and downstream homologous arms of the target gene as electroporated into the competent EGD-e cells. The *L. monocytogenes* transformants were grown at a non-permissive temperature of 42°C on BHI agar containing chloramphenicol to screen the homologous recombinant strain. This strain contained the pKSV7 recombinant plasmid integrated into its genome. To facilitate plasmid excision, the recombinant strains were passaged without antibiotics at a permissive temperature of 30°C. The deletion strains, which no longer carried the pKSV7 plasmid, were identified by their sensitivity to chloramphenicol. Confirmation of homologous gene exchange was achieved through PCR and sequencing analyses. For complementing the target gene in *L. monocytogenes* via homologous recombination, the integrative plasmid pIMK2 was utilized. The complementation plasmid was constructed by inserting the target gene along with its native promoter. This recombinant plasmid was then electroporated into competent *L. monocytogenes* cells that had the target gene deleted, which was confirmed through antibiotic resistance screening, PCR, and sequencing.

### Oxidative stress susceptibility assays

Three oxidants were employed to induce oxidative stress: Cu^2+^ as a lipid peroxidation inducer, H_2_O_2_ as an endogenous source of ROS, and diamide as a thiol-oxidizing agent that mimics oxygen exposure damage (23, 24). *L. monocytogenes* wild-type EGD-e, Δ*gshF,* and CΔ*gshF* strains were grown overnight at 37°C in BHI broth with shaking. The cultures were then diluted with 10 mmol/L phosphate-buffered saline (PBS; pH 7.4) to an absorbance of 0.6 at OD_600 nm_ [~2 × 10^9^ forming unit (cfu)/mL]. The bacterial suspension was serially diluted 10-fold and diluted six times. Next, 10 µL of each dilution was plated on BHI agar plates containing different concentrations of diamide (ranging from 1 to 2 mmol/L) and copper chloride (ranging from 0.25 to 1 mmol/L). Following incubation at 37°C for 24–48 hours, the growth of colonies on each plate was assessed and photographed. To assess susceptibility to H_2_O_2_, 0.1 mL portions of the cultures was spread onto BHI agar plates. Filter paper disks (diameter, 5 mm) soaked with 10 µL of 15% H_2_O_2_ were placed on the agar and incubated overnight.

### Proliferation of *L. monocytogenes* in RAW 264.7 macrophages

Intracellular propagation within macrophages was conducted using the following procedure. Overnight cultures of EGD-e, Δ*gshF,* and CΔ*gshF* strains were washed and suspended in 10 mmol/L PBS (pH 7.4). RAW 264.7 cells were cultured in Dulbecco's Modified Eagle Medium (DMEM) supplemented with 10% fetal bovine serum (FBS). The cells were then infected with bacteria at a multiplicity of infection (MOI) of 0.05 for 30 min. To eliminate extracellular bacteria, the infected cells were treated with DMEM containing 50 µg/mL gentamicin for an additional 30 minutes. Infected cells were subsequently incubated in DMEM supplemented with 50 µg/mL of gentamicin and 10% of FBS. At designated time points (2, 5, and 8 hours post-infection), the cells were washed with PBS and lysed by adding 300 µL of trypsin and 700 µL of ice-cold sterile distilled water to each well. Bacterial suspensions were then prepared by serially diluting them 10-fold and plating them on BHI agar plates for enumeration. To determine the proliferation rate, the total number of bacteria at each time point was divided by the total number of bacteria invading cells at 1 hour.

### Adhesion and invasion of *L. monocytogenes* in Caco-2 cells

To evaluate bacterial invasion and adhesion in human intestinal epithelial Caco-2 cells, the following steps were carried out. Overnight cultures of EGD-e, Δ*gshF,* and CΔ*gshF* strains were washed and suspended in 10 mmol/L PBS (pH 7.4). Monolayers of Caco-2 cells were cultured in DMEM containing 20% FBS. For the adhesion assay, the Caco-2 cells were infected with the bacterial strains at an MOI of 10 and incubated for 30 minutes. For the invasion assay, the Caco-2 cells were infected with the bacterial strains at an MOI of 10 and incubated for 90 minutes. Subsequently, the cells were treated with DMEM containing gentamicin at a concentration of 50 µg/mL for an additional 90 minutes to eliminate any remaining extracellular bacteria. After the incubation period, the cells were washed to remove residual antibiotics and lysed. Bacterial suspensions were then serially diluted and plated on BHI agar plates for the enumeration of CFUs. Adhesion was calculated as the ratio of the number of remaining colonies after washing with PBS to the number of colonies initially inoculated. On the other hand, invasion was defined as the ratio of the number of colonies recovered after gentamicin treatment to the number of colonies initially inoculated.

### Virulence of *L. monocytogenes* in the mouse model

The virulence of *L. monocytogenes* wild-type EGD-e, Δ*gshF,* and CΔ*gshF* strains was assessed in mice using the following procedure. Specifically, 6- to 8-week-old female ICR mice weighing 18–22 g were used for the animal infections. These mice were kept in a specific pathogen-free environment. Intraperitoneal injections were administered to each mouse with 10^6^ CFU of the respective *L. monocytogenes* strains. After 24 and 48 hours post-infection, the mice were humanely sacrificed. The liver and spleen of each animal were collected under aseptic conditions. The collected organs were homogenized in 10 mmol/L PBS (pH 7.4). The homogenates were then serially diluted, and the dilutions were plated on BHI agar plates to facilitate the enumeration of bacterial cells. A number of bacteria colonizing the organs was expressed as mean ± SEM of the log_10_CFU per organ for each group (eight mice).

### Transcriptomic profiles of *L. monocytogenes* exposed to oxidative stress

For the transcriptomic analysis of *L. monocytogenes* exposed to diamide and copper ions, the following procedure was conducted. Overnight cultures of wild-type EGD-e and Δ*gshF* strains were transferred to fresh BHI broth and allowed to grow with shaking at 37°C until the OD_600 nm_ reached 0.4. To induce exposure to diamide, the bacteria were treated with diamide at a final concentration of 2 mmol/L and further incubated at 37°C for 1 hour. For exposure to copper ions, the bacteria were treated with copper chloride at a final concentration of 1 mmol/L and further incubated at 37°C for 1 hour. After the incubation period, the bacteria were harvested by centrifugation. The harvested samples were then sent to Shanghai Majorbio Bio-Pharm Technology Co., Ltd. for RNA isolation, library construction, RNA sequencing, data processing, differential transcription analysis, and functional annotation. The procedures for these analyses were performed as previously described (25).

### Real-time quantitative PCR

Overnight cultures of wild-type *L. monocytogenes* EGD-e and Δ*gshF* strains were diluted with fresh BHI broth and recultured at 37°C until OD_600_ nm reached 0.4. The bacteria were treated with diamide at a final concentration of 2 mmol/L or copper chloride at a final concentration of 1 mmol/L and further incubated at 37°C for 1 hour. Total RNA was then extracted using the Column Bacterial total RNA Puriﬁcation Kit (Sangon), following the manufacturer’s instructions. To eliminate genomic DNA, DNase I (TaKaRa) was used, and cDNA was synthesized using reverse transcriptase (TOYOBO). For quantifying the mRNA levels of the target genes, quantitative real-time PCR (qRT-PCR) was conducted with cDNA samples, specific PCR primers (listed in Table S1), and SYBR quantitative PCR mix (TOYOBO). The mRNA levels of the target genes were normalized using the RNA polymerase beta subunit gene *rpoB* as an internal standard. Relative transcript levels were quantiﬁed using the 2^−ΔΔCT^ method and expressed as fold changes relative to the control condition.

### Detection of GshF regulation of *lmo1997-lmo2004* gene expression

The plasmid pAM401 was used to clone a fusion fragment containing the *lmo2004-lmo1997* promoter region and the *gfp* gene (P*_lmo2004_-gfp*). Subsequently, the recombinant plasmid was introduced into both the wild-type EGD-e strain and the Δ*gshF* deletion strain. All strains were initially cultured overnight in BHI broth at 37°C. The following day, they were diluted in fresh BHI broth and grown at 37°C until reaching an OD_600 nm_ of 0.4. At this point, copper chloride was added to the bacterial cultures to achieve a final concentration of 1 mmol/L, or diamide was added to the bacterial cultures to achieve a final concentration of 2 mmol/L, and the incubation continued at 37°C for an additional hour. Finally, the collected cultures were assayed using a microplate reader.

### Construction of the gene overexpression strain

The plasmid pAM401 was used to clone a fusion fragment, P*_dlt_-lmo2004-lmo1997*, which consisted of the promoter region of *dlt* and the *lmo2004-lmo1997* gene. This recombinant plasmid was introduced into the wild-type EGD-e strain through electroporation. The resulting strain, containing the recombinant plasmid, was designated as C*lmo1997-lmo2004*_P*_dlt_*. In addition, another fusion fragment, P*_dlt_-iiB^man^ (lmo2002*), containing the promoter regions of *dlt* and the *iiB^man^* gene, was cloned into the plasmid pAM401. Subsequently, the recombinant plasmid carrying the P*_dlt_-iiB^man^* fusion fragment was introduced into the wild-type EGD-e strain through electroporation. The strain carrying this recombinant plasmid was designated as C*iiB^man^_*P*_dlt_*.

### Statistical analyses

All experiments were replicated at least three times. Statistical analyses were performed using the software Prism9 (GraphPad Software). An unpaired two-tailed Student’s *t*-test was used to compare the means of the two groups. Differences with a calculated *P*-value above 0.05 were considered not significant, and statistically significant differences were noted: *P < 0.05, **P < 0.01, ***P < 0.001.

## RESULTS

### Deletion of *gshF* significantly decreased the tolerance of *L. monocytogenes* to copper ions

**Fig 1:**
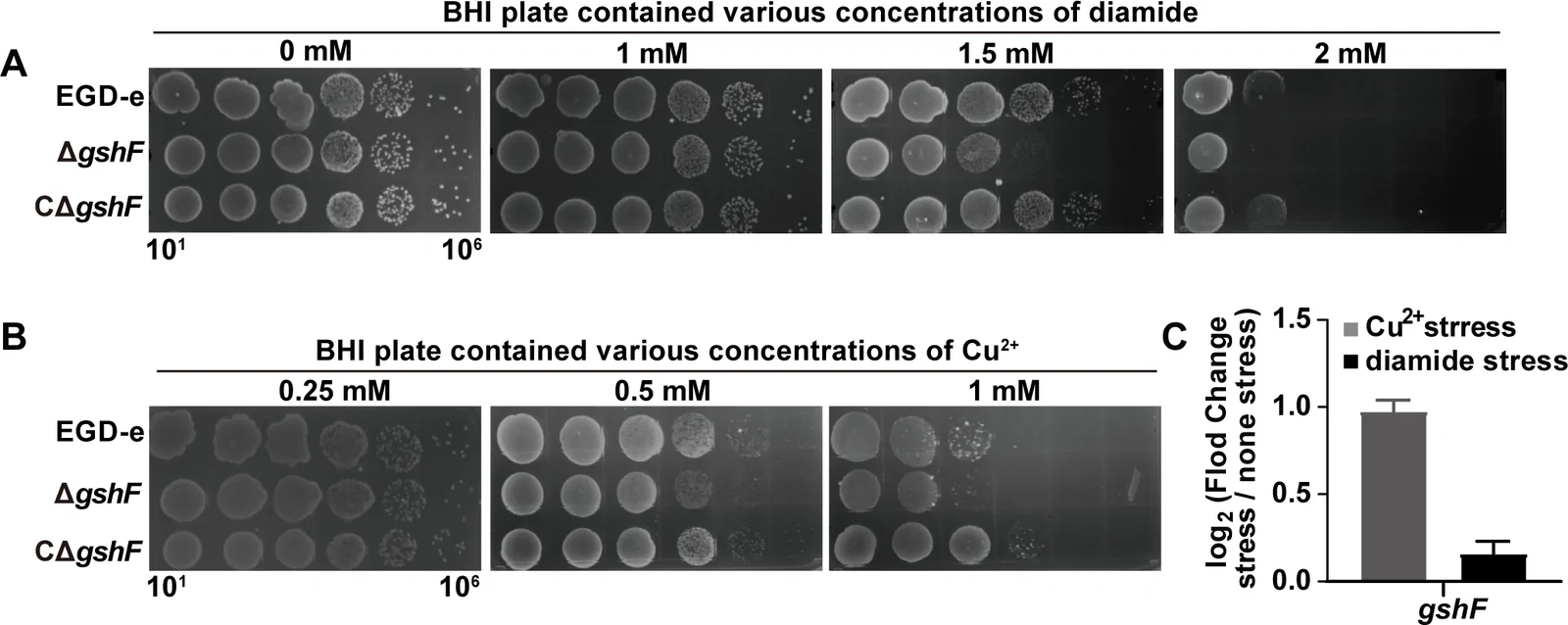
Deletion of *gshF* decreased the tolerance of *L. monocytogenes* to copper chloride and diamide. The survival of *L. monocytogenes* under oxidative stress conditions was investigated. Overnight cultures of the wild-type *L. monocytogenes* EGD-e, the *gshF* deletion strain Δ*gshF*, and the complement strain CΔ*gshF* were serially diluted and spotted onto BHI plates containing different concentrations of diamide (**A**) or Cu^2+^ (**B**). The plates were then incubated for 24–48 hours at 37°C. The data presented in the study are based on three replicates. (**C**) The transcription of the *gshF* gene in *L. monocytogenes* EGD-e and the *gshF* deletion strain was analyzed using qRT-PCR under stress conditions induced by 2 mmol/L diamide and 1 mmol/L copper chloride. The relative transcript levels were calculated as the mean ± SEM of the log_2_ (fold changes) from three replicates.

The *gshF* deletion strain exhibited a growth pattern similar to the reference strain EGD-e and the complemented strain CΔ*gshF* when cultured on BHI plates at 37°C (Fig. 1A). To assess the contribution of *gshF* to oxidative tolerance, *L. monocytogenes* EGD-e, Δ*gshF,* and CΔ*gshF* were exposed to various oxidative agents. The deletion of *gshF* resulted in decreased resistance to hydrogen peroxide (Fig. S1), diamide (Fig. 1A), and copper ions (Fig. 1B), with a reduction of 1–2 logs in bacterial survival upon exposure to different concentrations of diamide and copper ions (Fig. 1A and B). The complemented strain CΔ*gshF* exhibited resistance to diamide and copper ions similar to the wild-type strain (Fig. 1A and B). These results on hydrogen peroxide and diamide tolerance align with published data (19), and in addition, we demonstrated the role of GshF in copper ions tolerance. Furthermore, the transcription of *gshF* in the wild-type EGD-e strain was upregulated under diamide and copper ions stress (Fig. 1C).

### Deletion of *gshf* decreased the efficiency of bacterial invasion

**Fig 2:**
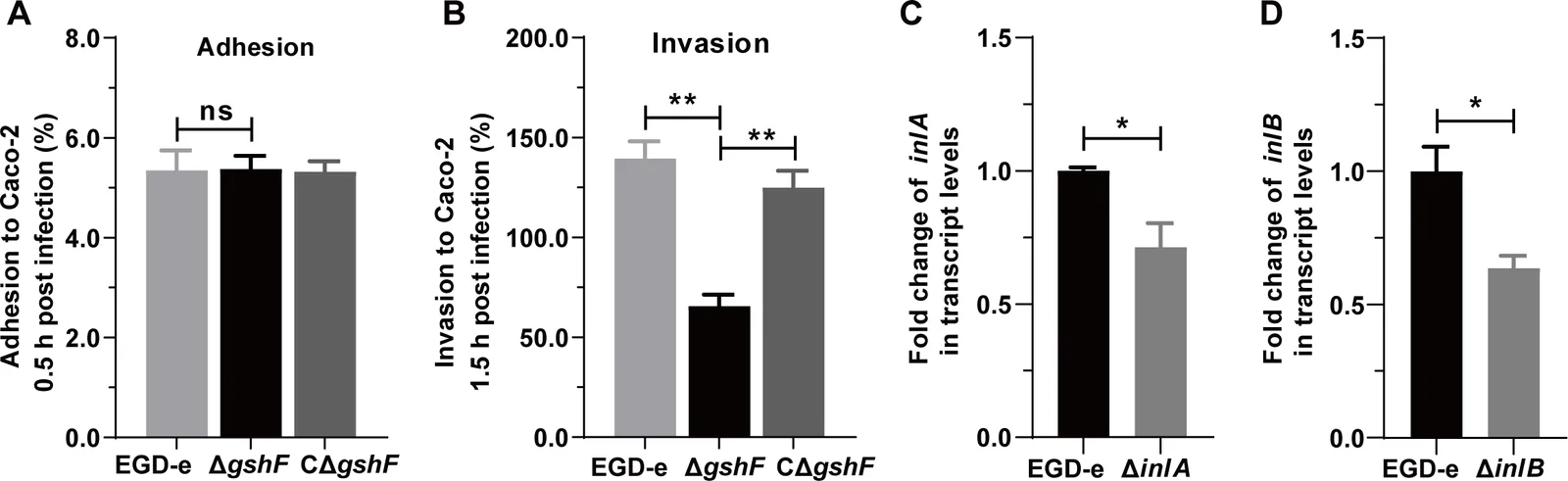
Deletion of *gshF* decreased the efficiency of bacterial invasion (**A and B**) To investigate the involvement of *gshF* in bacterial invasion and adhesion, Caco-2 epithelial cells were utilized. For adhesion assays, infected Caco-2 cells were lysed at 0.5 hour post-infection, and the resulting lysates were serially diluted and plated on BHI plates to determine viable bacterial counts. For invasion assays, the cell plates were incubated for 90 minutes, after which the supernatant was removed. The cells were then incubated with DMEM supplemented with gentamicin at a concentration of 50 µg/mL for an additional 90 minutes to eliminate any extracellular bacteria. Viable bacteria were subsequently serially diluted and plated on BHI plates. (**C and D**) Quantification of the mRNA levels of the *inlA* and *inlB* genes using qRT-PCR. Data are expressed as mean ± SEM of three replicates. ns, no significance; *, *P* < 0.05, **, *P* < 0.01.

To investigate the role of *gshF* in bacterial adhesion and invasion, Caco-2 epithelial cells were utilized to compare the adhesion and invasion abilities of the *gshF* deletion strain and the wild-type EGD-e strain. The results demonstrated that the *gshF* deletion strain displayed significantly reduced invasiveness, approximately 50% less than the wild-type strain, although the deletion did not have any noticeable impact on bacterial adhesion to the cells (Fig. 2A and B). Further analysis through qRT-PCR revealed that the transcript levels of the invasion-related proteins InlA and InlB decreased by approximately 30% and 35%, respectively, after the deletion of *gshF* (Fig. 2C and D). In addition, the deletion of *gshF* led to a decreased ability of EGD-e to proliferate within macrophages at 2 and 8 hours post-infection (Fig. S2A through D). Furthermore, mice infected with the *gshF* deletion strain exhibited lower bacterial loads compared to those infected with the EGD-e strain (Fig. S2E and F), consistent with 10403S as a model bacterium (22). Collectively, these findings indicate that *gshF* is necessary for bacterial invasion, proliferation, and virulence in mice.

### GshF altered global transcription profiles under oxidative stress, primarily affecting carbohydrate and amino acid metabolism

**Fig 3:**
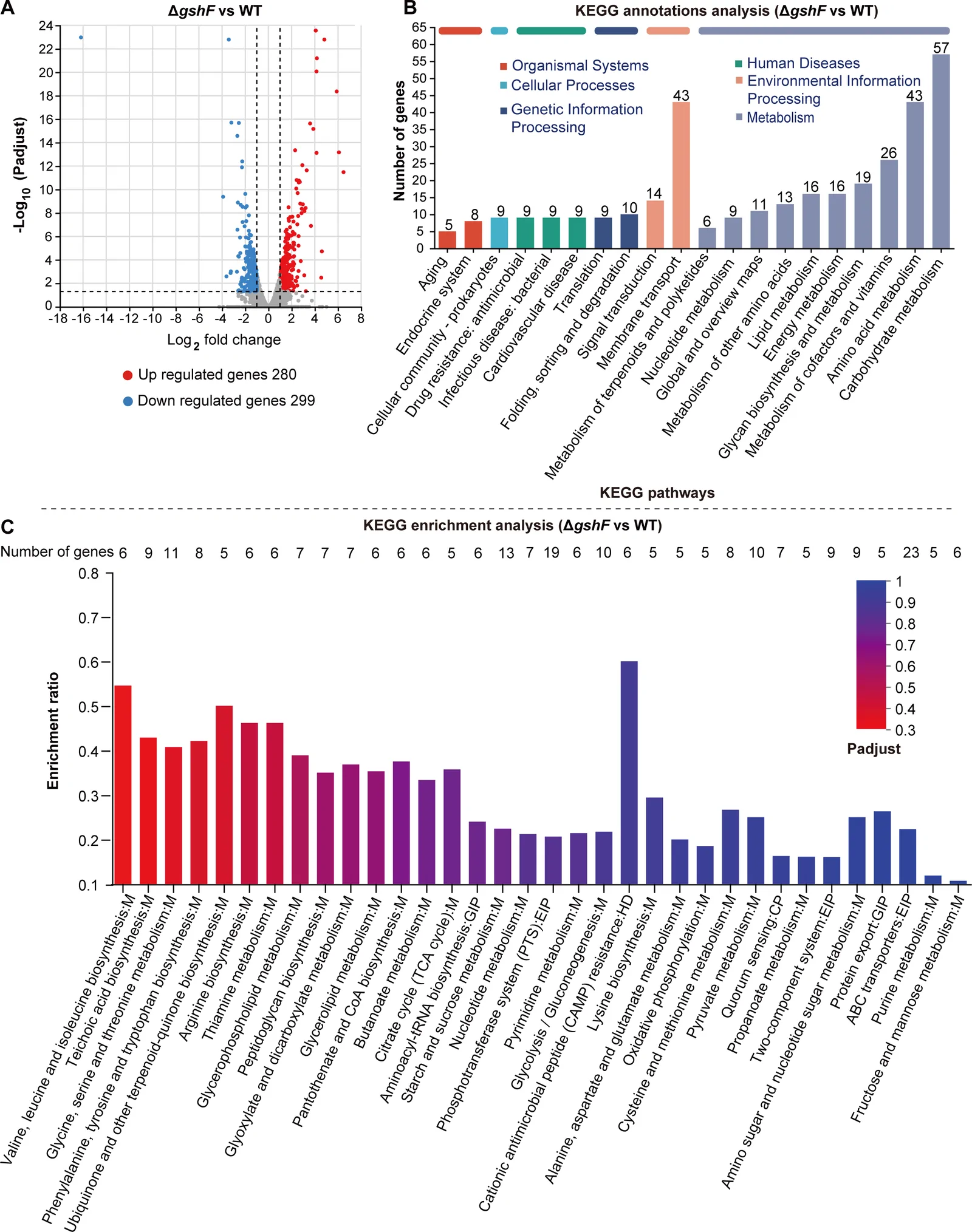
KEGG annotation and KEGG enrichment of the genes regulated by GshF in response to copper ions stress (**A**) The volcano plot illustrates the overall changes in transcript levels between the *gshF* deletion strain Δ*gshF* and the wild-type (WT) strain when grown in the presence of 1 mmol/L copper chloride. Genes with a log_2_ (fold change) greater than 1.0 or less than −1.0 and a significance level of *P* < 0.05 are indicated. (**B**) KEGG annotation analysis was performed to assess the functional implications of the genes regulated by GshF. The top 20 KEGG pathways in terms of abundance are presented. (**C**) KEGG enrichment analysis was performed to examine the functional implications of the genes regulated by GshF. The KEGG pathways were mapped, focusing on the target pathways where at least five genes were enriched.

**Fig 4:**
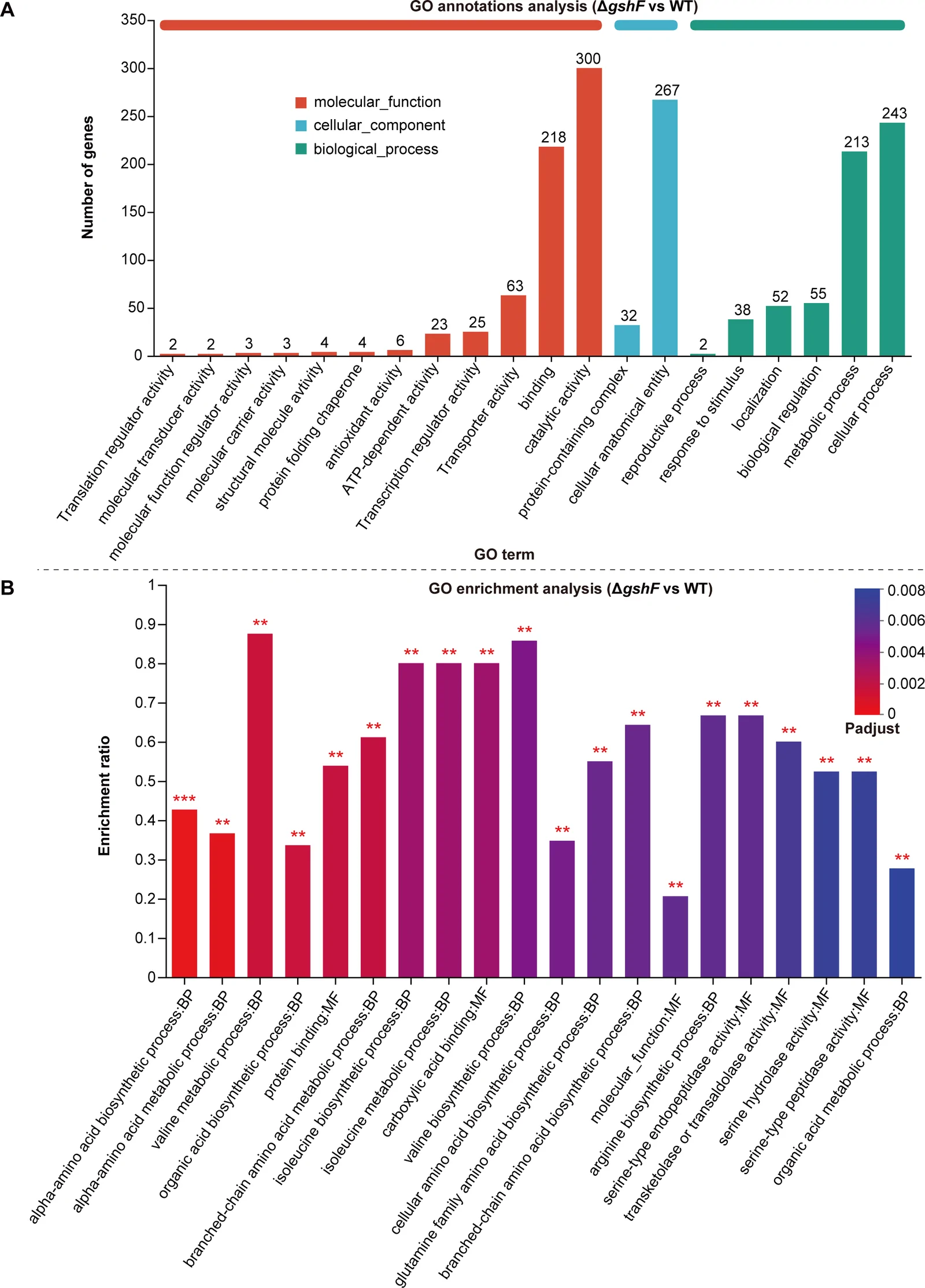
GO annotation and GO enrichment of the genes regulated by GshF in response to copper ions stress. (**A**) GO annotation analysis was conducted to evaluate the functional implications of the genes regulated by GshF. The top 20 GO terms in terms of abundance are presented. (**B**) GO enrichment analysis was performed to examine the functional implications of the genes regulated by GshF. Only the top 20 GO term enrichments with a significance level of *P*adjust less than or equal to 0.5 are displayed. Enrichments with a *P*adjust <0.01 are labeled **.

The transcriptomic analysis revealed significant differences in gene transcription between the Δ*gshF* and EGD-e strains under oxidative conditions. Specifically, 31 genes and 23 genes exhibited significantly higher or lower transcript levels, respectively, in the Δ*gshF* strain compared to EGD-e after exposure to 2 mmol/L diamide for 1 hour (Table S2). Similarly, under oxidative conditions induced by 1 mmol/L Cu^2+^ for 1 hour, 280 genes and 299 genes displayed significantly higher or lower transcript levels, respectively, in the Δ*gshF* strain compared to EGD-e (Table S2; Fig. 3A). After analyzing the differentially expressed genes, we conducted KEGG annotations analysis, which revealed significant associations with several biological pathways. Specifically, 56 genes were linked to carbohydrate metabolism, 43 to amino acid metabolism, 43 to membrane transport, 26 to the metabolism of cofactors and vitamins, and 19 to glycan biosynthesis and metabolism. These pathways ranked among the top five in terms of abundance among the 20 KEGG pathways we analyzed (Fig. 3B). Furthermore, our KEGG enrichment analysis highlighted specific pathways of interest. We found that 23 genes were linked to ABC transporters, 19 genes to the phosphotransferase system, 13 genes to starch and sucrose metabolism, 11 genes to glycine, serine, and threonine metabolism, 10 genes to glycolysis/gluconeogenesis, and 10 genes to pyruvate metabolism (Fig. 3C). In addition, we performed gene ontology (GO) annotations analysis, revealing the top five GO terms among the 20 most abundant GO terms were catalytic activity, cellular anatomical entity, cellular process, binding, and metabolic process (Fig. 4A). Moreover, our GO enrichment analysis revealed that the top 20 enriched GO terms were predominantly related to the biosynthesis and metabolic processes of multiple amino acids (Fig. 4B). Taken together, these findings suggest that the deletion of GshF significantly affects gene transcription related to carbohydrate metabolism and amino acid metabolism in response to oxidative resistance.

### Notable changes in transcript levels were observed in the PTS genes *lmo1997-lmo2004* in response to oxidative stress

**Fig 5:**
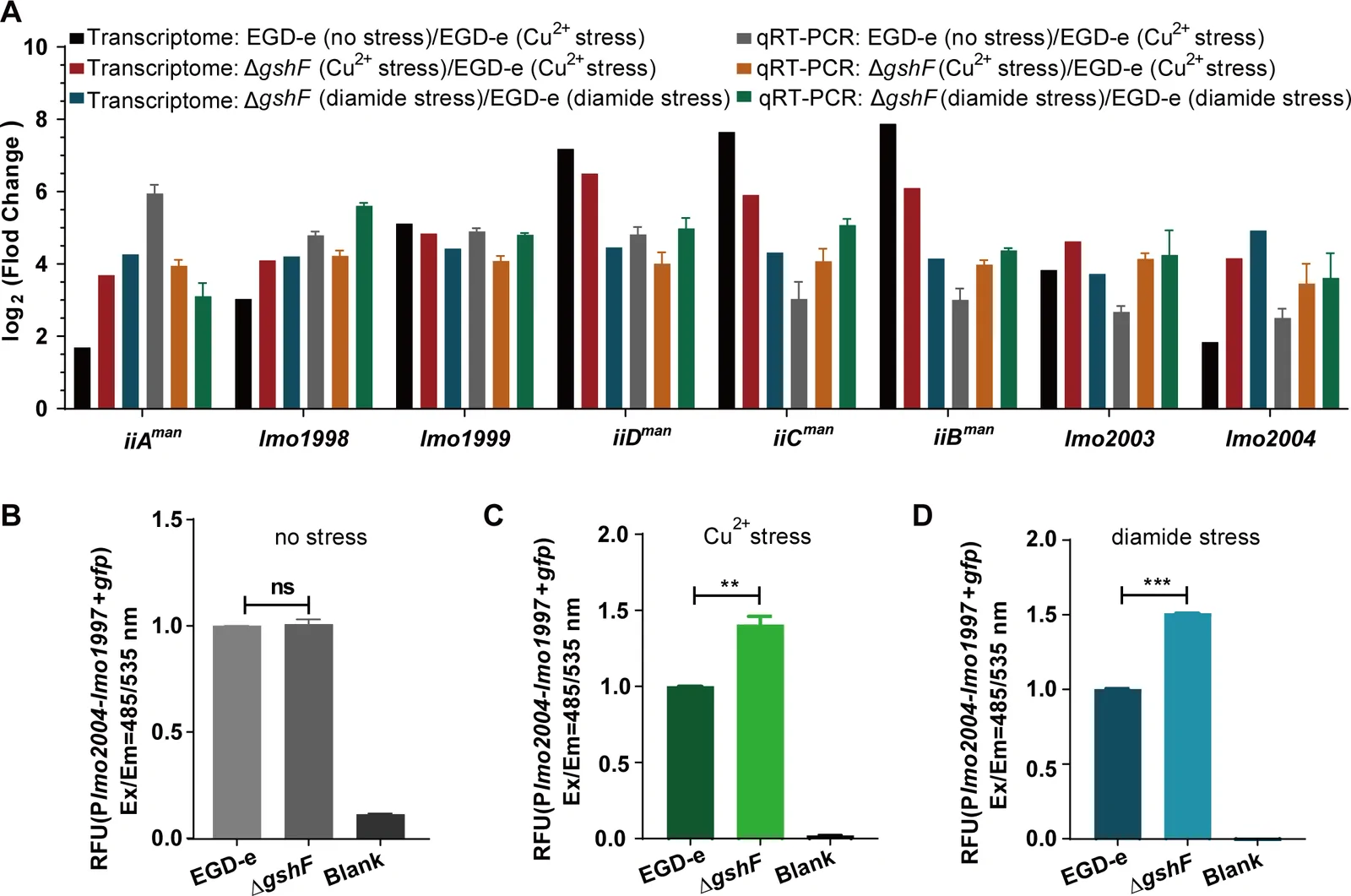
The PTS genes *lmo1997-lmo2004* were downregulated in wild-type EGD-e compared with *gshF* deletion strain in response to oxidative stress. (**A**) Transcriptomic data were obtained for the PTS genes *lmo1997-lmo2004* in both the wild-type *L. monocytogenes* EGD-e and the *gshF* deletion strain under 2 mmol/L diamide stress and 1 mmol/L copper chloride stress conditions. Subsequently, the transcription of the PTS genes *lmo1997-lmo2004* under 2 mmol/L diamide stress in the wild-type strain was validated using qRT-PCR. The relative transcript levels were calculated as the mean ± SEM of the log_2_ (fold changes) from three replicates. (**B – D**) A plasmid carrying the *lmo2004-lmo1997* promoter and *gfp* reporter gene was introduced into both EGD-e and the *gshF* deletion strain Δ*gshF*. The transcription of *lmo1997-lmo2004* in normal conditions and response to 1 mmol/L copper chloride stress and 2 mmol/L diamide stress was assessed by measuring the GFP expression using a microplate reader. Data are expressed as mean ± SEM of three replicates. ns, no significance; **P < 0.01, ***P < 0.001 .

KEGG enrichment analysis highlighted that 19 genes were associated with the phosphotransferase system (Table S2). The transcription levels of PTS genes *lmo1997-lmo2004* drew our attention to this study. Specifically, we observed that these genes were significantly upregulated in the Δ*gshF* strain compared to the wild-type strain under copper ions and diamide stress conditions, with more than 10-fold changes (Table S2; Fig. 5A). Interestingly, in response to copper ions stress, the wild-type strain exhibited transcriptional downregulation of the *lmo1997-lmo2004* genes compared to normal culture conditions (Table 1; Table S2). The qRT-PCR analysis confirmed the consistent transcription pattern observed in the transcriptomic results (Fig. 5A). Furthermore, the study observed that the promoter activity of P*_lmo2004_-gfp* was comparable in the Δ*gshF* strain and the wild-type strain under normal conditions (Fig. 5B). However, the promoter activity of P*_lmo2004_-gfp* was significantly higher in the Δ*gshF* strain compared to the wild-type strain under Cu^2+^stress (Fig. 5C) and diamide stress (Fig. 5D). These findings indicate that GshF regulates the transcription of PTS genes under oxidative stress conditions. In addition, several genes involved in thiol:disulfide redox metabolism showed upregulation, including *lmo0964* (*yjbH*), *lmo1059*, *lmo1233* (*trxA*), *lmo1609*, *lmo1860*, *lmo2152*, *lmo2393*, *lmo2426*, *lmo2478*, and *lmo2830* (Table 2; Table S2). Overall, these results suggest that GshF plays a role in bacterial oxidative resistance by influencing gene transcription related to PTS genes and thiol:disulfide redox metabolism-related genes.

**TABLE 1:** The transcription of PTS genes differs between the EGD-e and ΔgshF strains under stress conditions, as well as under stress conditions and normal conditions in the EGD-e strain^*a*^

	Oxidative stress of Cu2+					
Gene ID	log_2_fc（ΔgshF_stress/EGD-e_stress）	Significant/regulate	log_2_fc（EGD-e_stress/EGD- e_no stress）	Significant/regulate	Gene name	Gene description
lmo0021	–	–	−3.45	Yes/down	lmo0021	PTS fructose transporter subunit IIA
lmo0022	–	–	−4.88	Yes/down	lmo0022	PTS fructose transporter subunit IIB
lmo0023	–	–	−6.85	Yes/down	lmo0023	PTS fructose transporter subunit IIC
lmo0024	–	–	−8.35	Yes/down	lmo0024	PTS mannose transporter subunit IID
lmo0027	–	–	−6.99	Yes/down	lmo0027	PTS beta-glucoside transporter subunit IIABC
lmo0034	–	–	−2.34	Yes/down	lmo0034	PTS cellbiose transporter subunit IIC
lmo0096	–	–	−2.26	Yes/down	lmo0096	PTS mannose transporter subunit IIAB
lmo0097	–	–	−3.61	Yes/down	lmo0097	PTS mannose transporter subunit IIC
lmo0098	–	–	−3.70	Yes/down	lmo0098	PTS mannose transporter subunit IID
lmo0298	–	–	−4.86	Yes/down	lmo0298	PTS beta-glucoside transporter subunit IIC
lmo0299	–	–	−4.42	Yes/down	lmo0299	PTS beta-glucoside transporter subunit IIB
lmo0301	–	–	–	–	lmo0301	PTS beta-glucoside transporter subunit IIA
lmo0357	–	–	–	–	lmo0357	PTS sugar transporter subunit IIA
lmo0358	–	–	−1.89	Yes/down	lmo0358	PTS fructose transporter subunit IIBC
lmo0373	–	–	−2.47	Yes/down	lmo0373	PTS beta-glucoside transporter subunit IIC
lmo0374	–	–	−2.43	Yes/down	lmo0374	PTS beta-glucoside transporter subunit IIB
lmo0398	−3.27	Yes/down	−3.27	Yes/down	lmo0398	PTS sugar transporter subunit IIA
lmo0399	–	–	−7.90	Yes/down	lmo0399	PTS fructose transporter subunit IIB
lmo0400	–	–	−9.39	Yes/down	lmo0400	PTS fructose transporter subunit IIC
lmo0426	1.75	Yes/up	−2.63	Yes/down	lmo0426	PTS fructose transporter subunit IIA
lmo0427	1.65	Yes/up	–	–	lmo0427	PTS fructose transporter subunit IIB
lmo0428	1.95	Yes/up	−2.82	Yes/down	lmo0428	PTS fructose transporter subunit IIC
lmo0503	–	–	–	–	lmo0503	PTS fructose transporter subunit IIA
lmo0507	–	–	−3.47	Yes/down	lmo0507	PTS galactitol transporter subunit IIB
lmo0508	–	–	−1.49	Yes/down	lmo0508	PTS galactitol transporter subunit IIC
lmo0542	–	–	−3.66	Yes/down	lmo0542	PTS sorbitol transporter subunit IIA
lmo0543	–	–	−3.25	Yes/down	lmo0543	PTS sorbitol transporter subunit IIBC
lmo0544	–	–	−4.74	Yes/down	lmo0544	PTS sorbitol transporter subunit IIC
lmo0631	–	–	−6.83	Yes/down	lmo0631	PTS fructose transporter subunit IIA
lmo0632	–	–	−3.13	Yes/down	lmo0632	PTS fructose transporter subunit IIC
lmo0633	–	–	–	–	lmo0633	PTS fructose transporter subunit IIB
lmo0738	–	–	–	–	lmo0738	PTS beta-glucoside transporter subunit IIABC
lmo0781	–	–	−1.33	Yes/down	lmo0781	PTS mannose transporter subunit IID
lmo0782	–	–	−3.54	Yes/down	lmo0782	PTS mannose transporter subunit IIC
lmo0783	–	–	−1.43	Yes/down	lmo0783	PTS mannose transporter subunit IIB
lmo0784	–	–	−1.99	Yes/down	lmo0784	PTS mannose transporter subunit IIB
lmo0874	–	–	−5.33	Yes/down	lmo0874	PTS sugar transporter subunit IIA
lmo0875	–	–	−3.59	Yes/down	lmo0875	PTS beta-glucoside transporter subunit IIB
lmo0876	–	–	−4.09	Yes/down	lmo0876	PTS sugar transporter subunit IIC
lmo0901	–	–	−2.01	Yes/down	lmo0901	PTS cellbiose transporter subunit IIC
lmo0914	–	–	−7.89	Yes/down	lmo0914	PTS sugar transporter subunit IIB
lmo0915	–	–	−6.02	Yes/down	lmo0915	PTS sugar transporter subunit IIC
lmo0916	–	–	−4.88	Yes/down	lmo0916	PTS sugar transporter subunit IIA
lmo1002	–	–	–	–	ptsH	Phosphocarrier protein HPr
lmo1003	–	–	–	–	lmo1003	Phosphotransferase system enzyme I
lmo1017	−1.24	Yes/down	–	–	lmo1017	PTS glucose transporter subunit IIA
lmo1035	–	–	–	–	lmo1035	PTS beta-glucoside transporter subunit IIABC
lmo1095	–	–	−3.29	Yes/down	lmo1095	PTS cellbiose transporter subunit IIB
lmo1255	1.52	Yes/up	−4.14	Yes/down	lmo1255	PTS trehalose transporter subunit IIBC
lmo1719	−1.64	Yes/down	–	–	lmo1719	PTS lichenan transporter subunit IIA
lmo1720	−2.29	Yes/down	2.06	Yes/up	lmo1720	PTS lichenan transporter subunit IIB
lmo1971	–	–	−3.10	Yes/down	ulaA	PTS ascorbate transporter subunit IIC
lmo1972	–	–	–	–	lmo1972	PTS pentitol transporter subunit IIB
lmo1973	–	–	–	–	lmo1973	PTS sugar transporter subunit IIA
lmo1997	3.67	Yes/up	–	–	lmo1997	PTS mannose transporter subunit IIA
lmo2000	6.48	Yes/up	−7.17	Yes/down	lmo2000	PTS mannose transporter subunit IID
lmo2001	5.88	Yes/up	−7.63	Yes/down	lmo2001	PTS mannose transporter subunit IIC
lmo2002	6.08	Yes/up	−7.85	Yes/down	lmo2002	PTS mannose transporter subunit IIB
lmo2096	–	–	−2.36	Yes/down	lmo2096	PTS galacticol transporter subunit IIC
lmo2097	–	–	−3.85	Yes/down	lmo2097	PTS galacticol transporter subunit IIB
lmo2098	–	–	–	–	lmo2098	PTS galacticol transporter subunit IIA
lmo2135	–	–	−2.61	Yes/down	lmo2135	PTS fructose transporter subunit IIC
lmo2136	–	–	–	–	lmo2136	PTS fructose transporter subunit IIB
lmo2137	–	–	–	–	lmo2137	PTS fructose transporter subunit IIA
lmo2259	–	–	3.02	Yes/up	lmo2259	PTS beta-glucoside transporter subunit IIA
lmo2335	–	–	−5.14	Yes/down	fruA	PTS fructose transporter subunit IIABC
lmo2373	1.30	Yes/up	–	–	lmo2373	PTS beta-glucoside transporter subunit IIB
lmo2649	–	–	−5.48	Yes/down	ulaA	PTS system ascorbate transporter subunit IIC
lmo2650	–	–	−5.75	Yes/down	lmo2650	MFS transporter
lmo2651	−2.43	Yes/down	−5.06	Yes/down	lmo2651	PTS mannitol transporter subunit IIA
lmo2665	–	–	−2.15	Yes/down	lmo2665	PTS galacticol transporter subunit IIC
lmo2666	-	–	−3.38	Yes/down	lmo2666	PTS galacticol transporter subunit IIB
lmo2667	–	–	−2.68	Yes/down	lmo2667	PTS galacticol transporter subunit IIA
lmo2683	−3.42	Yes/down	−1.46	Yes/down	lmo2683	PTS cellbiose transporter subunit IIB
lmo2684	−1.93	Yes/down	−3.90	Yes/down	lmo2684	PTS cellbiose transporter subunit IIC
lmo2685	−1.13	Yes/down	−4.83	Yes/down	lmo2685	PTS cellbiose transporter subunit IIA
lmo2708	−2.68	Yes/down	−7.10	Yes/down	lmo2708	PTS cellbiose transporter subunit IIC
lmo2733	1.13	Yes/up	−1.24	Yes/down	lmo2733	PTS fructose transporter subunit IIABC
lmo2762	–	–	−2.93	Yes/down	lmo2762	PTS cellbiose transporter subunit IIB
lmo2763	–	–	−2.32	Yes/down	lmo2763	PTS cellbiose transporter subunit IIC
lmo2765	–	–	−2.78	Yes/down	lmo2765	PTS cellbiose transporter subunit IIA
lmo2772	–	–	−3.43		lmo2772	PTS beta-glucoside transporter subunit IIABC
lmo2780	–	–	–	–	lmo2780	PTS cellbiose transporter subunit IIA
lmo2782	–	–	−4.30	Yes/down	lmo2782	PTS cellbiose transporter subunit IIB
lmo2783	–	–	−3.66	Yes/down	lmo2783	PTS cellbiose transporter subunit IIC
lmo2787	–	–	−4.02	Yes/down	bvrB	Beta-glucoside-specific phosphotransferase enzyme II ABC component
lmo2797	–	–	−2.71	Yes/down	lmo2797	PTS mannitol transporter subunit IIA
lmo2799	–	–	−2.55	Yes/down	lmo2799	PTS mannitol transporter subunit IIBC

^*a*^ "–" means that the genes listed in the table did not show significant differences in transcript levels.

**TABLE 2:** The transcription of thiol:disulfide redox metabolism-related genes differs between the EGD-e and ΔgshF strains under stress conditions, as well as under stress conditions and normal conditions in EGD-e strain^*a*^

	Oxidative stress of Cu2+					
Gene ID	Log_2_fc（ΔgshF_stress/EGD-e_stress）	Significant/regulate	Log_2_fc（EGD-e_stress/EGD-e_non stress）	Significant/regulate	Gene name	Gene description
lmo0964	1.37	Yes/up	-	-	yjbH	Hypothetical protein
lmo1059	1.70	Yes/up	1.44	Yes/up	lmo1059	Hypothetical protein
lmo1233	1.71	Yes/up	-	-	trxA	Thioredoxin
lmo1609	1.86	Yes/up	-	-	lmo1609	Thioredoxin
lmo1860	1.04	Yes/up	−1.19	Yes/down	lmo1860	Methionine sulfoxide reductase A
lmo1903	-	-	-	-	lmo1903	Thioredoxin
lmo2152	1.05	Yes/up	-	-	lmo2152	Thioredoxin
lmo2191	-	-	2.26	Yes/up	spxA	ArsC family transcriptional regulator
lmo2344	-	-	-	-	lmo2344	Hypothetical protein
lmo2393	2.15	Yes/up	-	-	lmo2393	Hypothetical protein
lmo2424	-	-	2.18	Yes/up	lmo2424	Thioredoxin
lmo2426	1.60	Yes/up	1.96	Yes/up	lmo2426	Hypothetical protein
lmo2478	1.05	Yes/up	-	-	lmo2478	Thioredoxin reductase
lmo2830	2.44	Yes/up	-	-	lmo2830	Thioredoxin

^*a*^ "-" means that the genes listed in the table did not show significant differences in transcript levels.

### Overexpression of *lmo1997-lmo2004* decreased oxidative tolerance to copper ions and diamide

**Fig 6:**
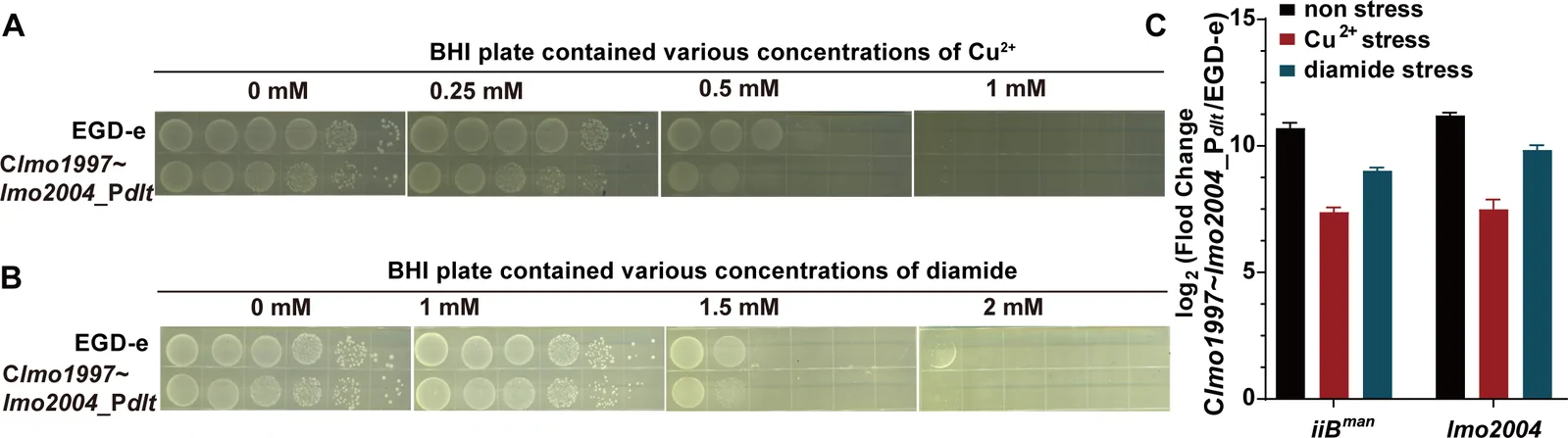
Overexpression of *lmo1997-lmo2004* decreased oxidative tolerance of *L. monocytogenes* to copper chloride and diamide. To investigate the survival of *L. monocytogenes* under oxidative stress conditions, overnight cultures of the wild-type strain *L. monocytogenes* EGD-e and the *lmo1997-lmo2004* overexpression strain C*lmo1997-lmo2004_P_dlt_* were serially diluted and spotted onto BHI plates containing various concentrations of Cu^2+^ (**A**) and diamide (**B**). The plates were then incubated at 37°C for 24–48 hours. The data presented in the study are based on three replicates. (**C**) The transcription levels of *lmo1997-lmo2004* were determined using qRT-PCR in both the wild-type *L. monocytogenes* and the *lmo1997-lmo2004* overexpression strain C*lmo1997-lmo2004_P_dlt_* under stress conditions induced by 2 mmol/L diamide and 1 mmol/L copper chloride. The relative transcript levels were calculated as the mean ± SEM of the log_2_ (fold changes) from three replicates.

To investigate the impact of *lmo1997-lmo2004* overexpression on oxidative tolerance, both the wild-type EGD-e and the *lmo1997-lmo2004* overexpression strain, C*lmo1997-lmo2004*_P*_dlt_*, were exposed to various oxidative agents. Overexpression of *lmo1997-lmo2004* in EGD-e led to a decreased resistance to copper ions, resulting in a 2-log reduction in bacterial survival when exposed to 0.5 mmol/L copper chloride (Fig. 6A). Similarly, overexpression of *lmo1997-lmo2004* reduced resistance to diamide, with a 1-log decrease in bacterial survival when exposed to concentrations of 1.5 mmol/L and 2 mmol/L diamide (Fig. 6B). Transcriptional activity of the *iiB^man^* and *lmo2004* genes was observed, indicating operon transcription. Notably, the transcription of the *iiB^man^* gene in C*lmo1997-lmo2004_P_dlt_* strain exhibited a significant upregulation, with fold changes of 1678, 166, and 518 compared to the wild-type strain under normal conditions, 1 mmol/L copper ions stress, and 2 mmol/L diamide stress, respectively (Fig. 6C). Similarly, for another gene in this operon, *lmo2004*, the transcription levels were upregulated by 2372, 183, and 923-fold, respectively (Fig. 6C). These findings indicate that upregulation of *lmo1997-lmo2004* renders *L. monocytogenes* less resistant to copper ions and diamide oxidative stress.

### Deletion of *iiB^man^* increased the oxidative tolerance to copper ions

**Fig 7:**
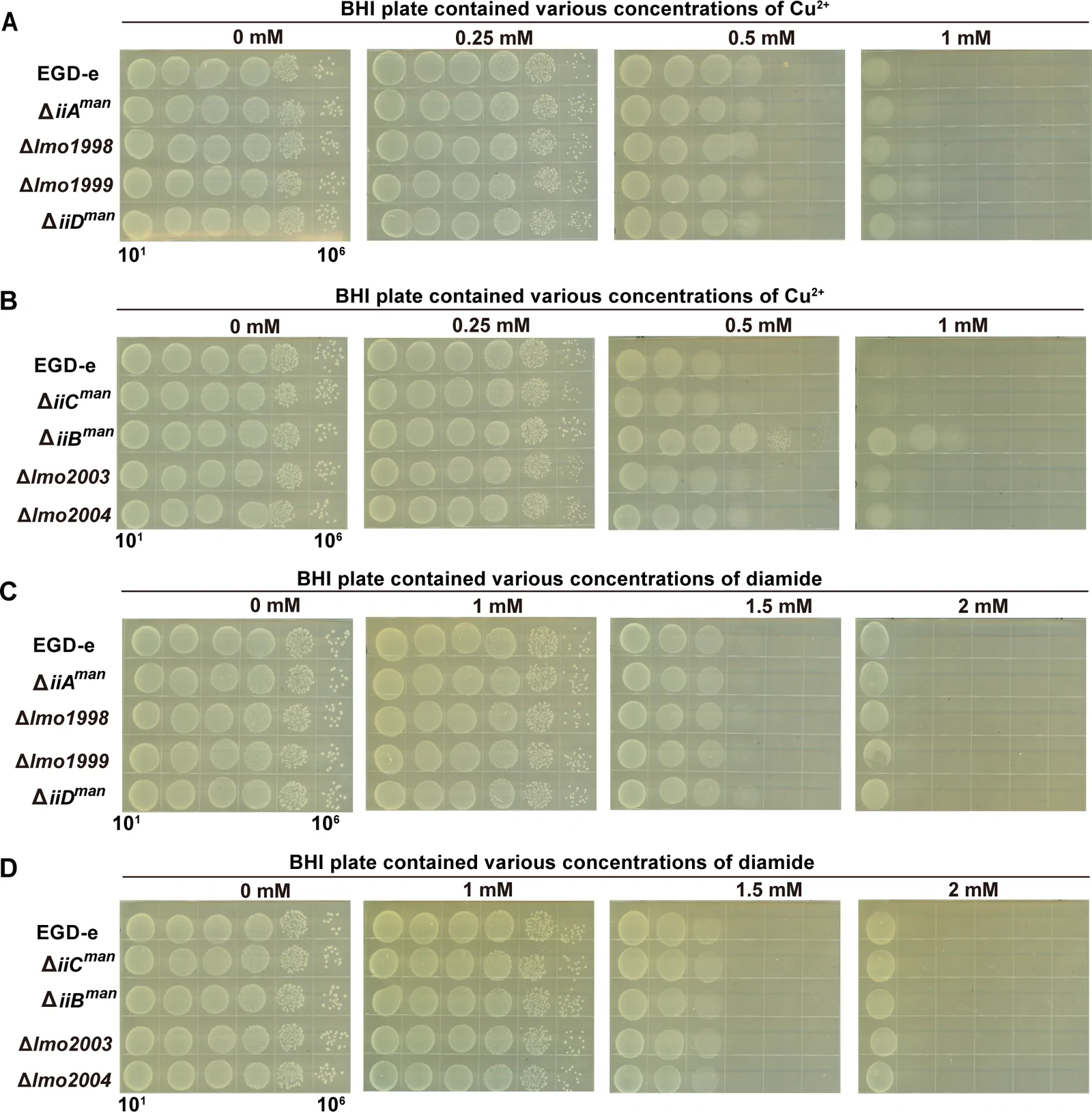
Deletion of *iiB^man^* increased *L. monocytogenes* oxidative tolerance to copper chloride. The survival of *L. monocytogenes* under oxidative stress conditions was assessed. Overnight cultures of the wild-type strain *L. monocytogenes* EGD-e and deletion strains Δ*iiA^man^*, Δ*lmo1998*, Δ*lmo1999*, and Δ*iiD^man^* were serially diluted and spotted onto BHI plates containing various concentrations of Cu^2+^ (**A**) and diamide (**C**). Similarly, EGD-e and deletion strains Δ*iiC^man^*, Δ*iiB^man^*, Δ*lmo2003*, and Δ*lmo2004* were grown overnight, serially diluted, and spotted onto BHI plates containing various concentrations of Cu^2+^ (**B**) and diamide (**D**). All strains were then incubated at 37°C for 24–48 hours. Data are based on three replicates. The data presented in the study are based on three replicates.

To identify which gene within the *lmo1997-lmo2004* operon contributes to the decreased resistance to oxidative tolerance in *L. monocytogenes*, gene deletion strains were generated for each individual gene in the operon. The wild-type *L. monocytogenes* strain and the deletion strains Δ*iiA^man^ (lmo1997*), Δ*lmo1998*, Δ*lmo1999*, Δ*iiD^man^ (lmo2000*), Δ*iiC^man^ (2001*), Δ*iiB^man^ (lmo2002*), Δ*lmo2003*, and Δ*lmo2004* were exposed to copper ions and diamide to assess their resistance. Interestingly, the wild-type strain EGD-e and the deletion strains *iiA^man^*, *lmo1998*, *lmo1999*, *iiD^man^*, *iiC^man^*, *iiB^man^*, *lmo2003,* and *lmo2004* exhibited similar resistance to diamide (Fig. 7C and D). Conversely, among the gene deletions investigated, only the deletion of *iiB^man^* resulted in increased resistance to copper ions compared to the wild-type strain EGD-e, as indicated by a higher number of surviving bacteria (Fig. 7A and B). However, the deletion of *iiA^man^*, *lmo1998*, *lmo1999*, *iiD^man^*, *iiC^man^*, *lmo2003,* and *lmo2004* exhibited similar growth patterns compared to the strain EGD-e. These findings suggest that *iiB^man^* is the critical gene within the *lmo1997-lmo2004* operon that renders *L. monocytogenes* less resistant to oxidative stress induced by copper ions.

### Overexpression of *iiB^man^* decreased oxidative tolerance of *L. monocytogenes* to copper ions

**Fig 8:**
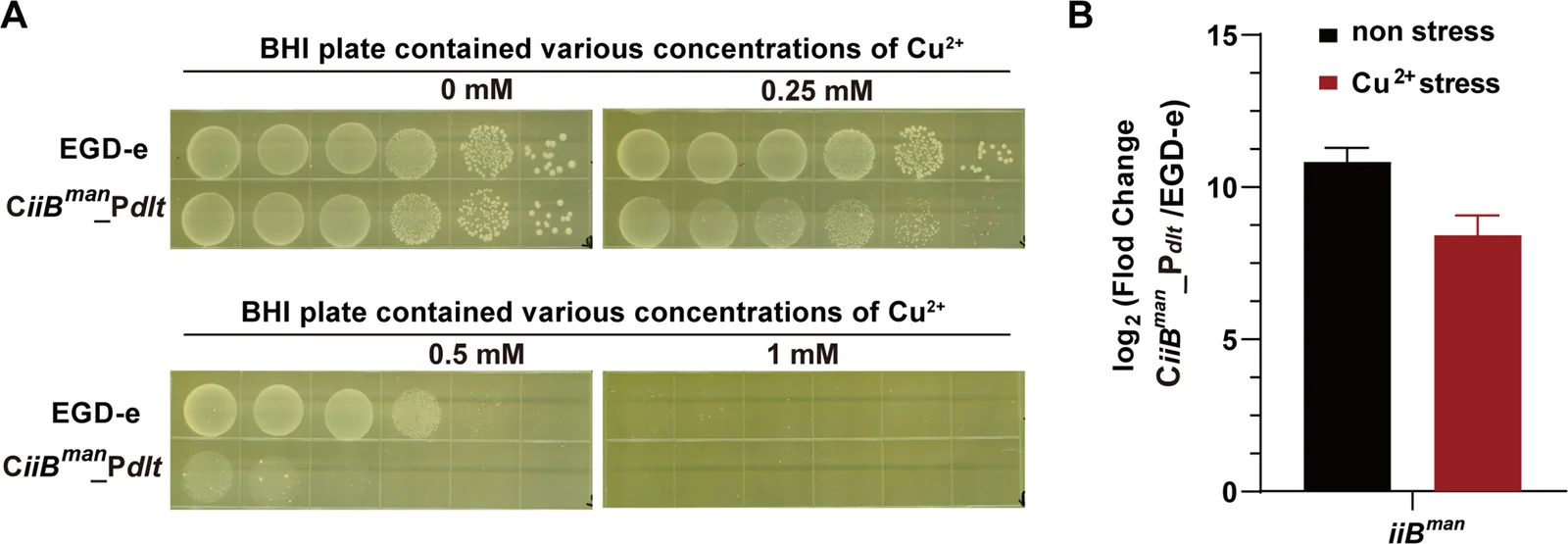
Overexpression of *iiB^man^* decreased oxidative tolerance of *L. monocytogenes* to copper chloride. (**A**) Overnight cultures of the wild-type strain EGD-e and the *iiB^man^* overexpression strain C*iiB^man^_P_dlt_* were serially diluted and spotted onto BHI plates containing various concentrations of Cu^2+^. The plates were then incubated at 37°C for 24–48 hours. The data presented in the study are based on three replicates. (**B**) The transcription levels of *iiB^man^* were assessed using qRT-PCR in both the wild-type *L. monocytogenes* EGD-e and the *iiB^man^* overexpression strain C*iiB^man^_P_dlt_* under the presence of 1 mmol/L copper chloride. The relative transcript levels were calculated as the mean ± SEM of the log_2_ (fold changes) from three replicates.

To investigate the impact of *iiB^man^* overexpression on oxidative tolerance in *L. monocytogenes*, both the wild-type strain and the C*iiB^man^* _P*_dlt_* strain were exposed to copper chloride. The results revealed that overexpression of *iiB^man^* in C*iiB^man^* _P*_dlt_* led to a decreased resistance to copper chloride compared to the wild-type strain, with a 3-log reduction in bacterial survival (Fig. 8A). Furthermore, the transcription of *iiB^man^* in C*iiB^man^* _P*_dlt_* was significantly upregulated compared to the wild-type *L. monocytogenes* EGD-e, with a 1863-fold higher transcription in the normal culture condition and a 359-fold higher transcription under 1 mmol/L copper ions stress (Fig. 8B). These findings consistently suggest that the overexpression of IIB^man^ (Lmo2002) renders *L. monocytogenes* less resistant to the oxidative stress induced by copper ions.

## DISCUSSION

Glutathione is a prominent low molecular weight thiol found in various living organisms (9). In bacteria, it plays a crucial role in multiple metabolic processes, including thiol redox homeostasis, defense against oxygen toxicity, and protein folding (26). *L. monocytogenes* possesses the GshF responsible for glutathione synthesis (19). Recent studies have emphasized the significance of GSH in regulating the virulence of *L. monocytogenes*. Upon entering the host cell cytosol, this facultative intracellular pathogen coordinates the expression of numerous essential virulence factors by allosterically binding GSH to the Crp-Fnr family transcriptional regulator PrfA (20). GSH can reversibly inhibit the activity of the pore-forming virulence factor listeriolysin O through naturally occurring S-glutathionylation (21). The regulation of PrfA and LLO by GSH provides a preventive strategy against *Listeria* infection, and targeting *gshF* may serve as an approach to attenuate *Listeria* virulence. Our investigation revealed that the absence of *gshF* led to reduced efficiency of *L. monocytogenes* EGD-e in invading host cells during infection, which captured our attention. *L. monocytogenes* employs internalin A (InlA) and internalin B (InlB), two molecules involved in surface adhesion and invasion, to enter the host cell (27, 28). By performing qRT-PCR analysis, we observed that the absence of *gshF* resulted in approximately 30% and 35% reduction in the transcription of *inlA* and *inlB*, respectively, while the overall invasiveness decreased by approximately 50%. Notably, previous studies have demonstrated that S-glutathionylation and allosteric regulation at a conserved cysteine residue post-translationally modifies LLO (21) and PrfA (20). Based on this knowledge, we formulated a hypothesis that InlB, which contains cysteine residues, may undergo reversible activation through natural S-glutathionylation or allosteric regulation by GSH.

The current understanding suggests that copper exerts a direct antibacterial effect and/or supports the antibacterial function of innate immune cells (29). Copper toxicity is mediated through the generation of ROS and its involvement in Fenton-like chemistry with ROS and RNS, leading to an increase in the production of reactive radicals and protein aggregation (30, 31). The utilization of copper as antimicrobial material or coating has gained attention due to concerns about antibiotic resistance and the need to reduce antibiotic usage (32). Copper resistance genes also serve as crucial virulence factors for bacterial pathogens (30). In the case of *S. pyogenes*, GSH plays a role in copper tolerance, allowing bacteria to maintain their metabolism even in the presence of excessive copper ions (17). In our study, we observed that the absence of *gshF* in *L. monocytogenes* EGD-e significantly reduced the pathogen’s tolerance to the copper oxidizing environment, indicating GSH plays a role in copper tolerance in *L. monocytogenes*. In addition, in the absence of *gshF*, genes involved in thiol:disulfide redox metabolism in *L. monocytogenes* were upregulated in response to copper ions and diamide. These genes are known to play a role in counteracting oxidative stress (33), indicating their involvement in compensating for the loss of GSH.

PTS is a bacterial multiprotein phosphorelay system that facilitates the transport of carbohydrates across the cytoplasmic membrane while phosphorylating them (34). Besides its role in sugar transport, certain PTSs have been implicated in bacterial resistance to oxidative stress, biofilm formation, virulence, and bacteriocin production (35 – 40). It has been observed that PTS can be induced by oxidative stress (41), and bacterial cells lacking a mannose-specific PTS face significant challenges in the energy generation processes required to mount an effective response to peroxide-induced stress, resulting in increased sensitivity to peroxides (42). Our transcriptomic analysis revealed that the presence of GshF in *L. monocytogenes* EGD-e negatively regulates several PTS genes under oxidative stress conditions (Table 1; Table S2). For instance, in response to copper ions stress, the transcription of PTS sugar transporter subunits IIA^man^ (Lmo1997), IIB^man^ (Lmo2002), IIC^man^ (Lmo2000), and IID^man^ (Lmo2001) within the *lmo1997-lmo2004* operon was significantly downregulated in the wild-type strain. However, in the *gshF* deletion strain, the transcription levels of these PTS genes were significantly upregulated in response to copper ions stress. This study represents the first report on the regulatory relationship between GshF and PTS in bacteria. We speculate that *gshF* is essential for the regulation of PTS gene transcription. As a glutathione synthase, GshF may not directly control the transcription of downstream genes. However, GSH synthesized by *gshF* could potentially play a critical role in modifying transcription factors that are involved in PTS gene regulation. Previous studies have shown that the *pts* operons *mptACD*, *mpoABCD,* and *lpo* are transcribed by the σ^54^-dependent RNA polymerase and their transcription is activated by ManR or LacR (38, 43, 44). These regulatory proteins belong to the LevR family of PRD (PTS regulation domain)-containing transcription activators of *B. subtilis* (45, 46). Furthermore, *in silico* analysis has identified putative PRD regulators of several *pts* operons of *L. monocytogenes*, including GntR, ManR, LicR, LacR, and MtlR (47). Notably, each of these regulators contains at least one cysteine residue. Considering the GSH’s potential to modify transcription factors with cysteine residues, a mechanism similar to that observed in the regulation of LLO (21) and PrfA (20) may be at play. Therefore, it is possible that GSH synthesized by *gshF* modifies these PRD regulators, thereby affecting their ability to regulate the transcription of PTS genes under oxidative stress conditions.

In this study, we observed that overexpression of *lmo1997-lmo2004* led to reduced oxidative tolerance under both copper ions and diamide stress conditions. However, only single deletion of *iiB^man^ in lmo1997-lmo2004* revealed a significant role in coping with copper ions stress, and its overexpression successfully reversed this phenotype. The presence of copper ions induces the generation of reactive radicals that lead to cellular damage by depleting enzyme activities through lipid peroxidation (23). Moreover, copper ions can bind to lipopolysaccharides or peptidoglycans, thereby affecting the stability of the bacterial cell envelope (32). On the other hand, diamide, a thiol-oxidizing agent, mimics the damage caused by oxygen exposure (24). Taken together, our results suggest that the mechanisms underlying bacterial responses to copper ions and diamide stress are distinctly different. However, the specific roles of the genes in the *lmo1997-lmo2004* cluster under oxidative stress conditions remain to be elucidated. For instance, the effects of overexpressing *iiB^man^* in response to diamide stress, as well as the roles of other genes in the operon during both copper and diamide stress, remain unexplored. Furthermore, *iiB^man^* is a component of the mannose-specific PTS system IIB (33, 47). It is crucial to gain a deeper understanding of the molecular mechanisms involving *iiB^man^* that *L. monocytogenes* utilizes to adapt to specific niche environments both outside and inside the host. In addition, in the absence of *gshF*, apart from the four PTS genes (*lmo1997*, *lmo2000*, *lmo2001*, and *lmo2002*) we focused on in this study, there are 15 PTS genes that exhibit significant changes in transcript levels under oxidative conditions induced by copper ions. These PTS genes include three *pts* operons (*lmo0426-lmo0428, lmo1719/lmo1720,* and *lmo2683-lmo2685*). Interestingly, *pts* operon *lmo1719/lmo1720* and *lmo2683-lmo2685* are regulated by the same transcription activator, LacR (47). Further research will delve into the specific roles of these PTS genes in responding to copper ions-induced stress and explore the regulatory interactions between GshF and these PTS genes.

In conclusion, our study provides evidence for the important biological role of GshF in bacterial environmental adaptation, particularly through its regulation of the PTS component. These speculations also highlight intriguing avenues for further exploration of the complex interplay between GSH, GshF, and the transcriptional regulation of PTS genes in *L. monocytogenes*. A deeper understanding of these mechanisms has the potential to elucidate the bacterium’s adaptive responses to environmental stress and its ability to cope with oxidative challenges, thereby providing valuable insights into potential targets for controlling *Listeria* infections.

## SUPPLEMENTAL MATERIAL

Figures S1 and S2, Tables S1 and S2 spectrum.02365-23-s0001.docx Figure S1: Deletion of *gshF* decreased the tolerance of *L. monocytogenes* to hydrogen peroxide; Figure S2: Deletion of *gshF* decreased the bacterial proliferation efficiency and the virulence in mice; Table S1: The primers used in this study; Table S2: The transcription of genes differs between EGD-e and ΔgshF strains under stress conditions, or under stress conditions and normal conditions in EGD-e strain, or ΔgshF and EGD-e under normal conditions.

ASM does not own the copyrights to Supplemental Material that may be linked to, or accessed through, an article. The authors have granted ASM a non-exclusive, world-wide license to publish the Supplemental Material files. Please contact the corresponding author directly for reuse.
