# Supplementary material for: Listeria monocytogenes GshF contributes to oxidative stress tolerance via regulation of the phosphoenolpyruvate-carbohydrate phosphotransferase system
Source: Microbiol Spectr. 2023 Sep 5;11(5):e02365-23. doi: 10.1128/spectrum.02365-23 (PMC10580955; doi:10.1128/spectrum.02365-23)
Supplement: Figures S1 and S2, Tables S1 and S2 — Figure S1: Deletion of gshF decreased the tolerance of L. monocytogenes to hydrogen peroxide; Figure S2: Deletion of gshF decreased the bacterial proliferation efficiency and the virulence in mice; Table S1: The primers used in this study; Table S2: The transcription of genes differs between EGD-e and ΔgshF strains under stress conditions, or under stress conditions and normal conditions in EGD-e strain, or ΔgshF and EGD-e under normal conditions. [file spectrum.02365-23-s0001.docx]

**Supplemental Information for:**

***Listeria monocytogenes* GshF contributes to oxidative stress tolerance via regulation of the phosphoenolpyruvate-carbohydrate phosphotransferase system**

Mianmian Chen^1^^, 2, 3, 4#^, Jiaxue Zhang^1, 2, 3, 4#^, Jing Xia^1, 2, 3, 4#^, Jing Sun^1, 2, 3, 4^, Xian Zhang^1, 2, 3, 4^, Jiali Xu^1, 2, 3, 4^, Simin Deng^1, 2, 3, 4^, Yue Han^1, 2, 3, 4^, Lingli Jiang^5^, Houhui Song^1, 2, 3, 4^*, Changyong Cheng^1, 2, 3, 4^*

^1^Key Laboratory of Applied Technology on Green-Eco-Healthy Animal Husbandry of Zhejiang Province, College of Animal Science and Technology & College of Veterinary Medicine, Zhejiang A&F University, Hangzhou, China

^2^Zhejiang Provincial Engineering Research Center for Animal Health Diagnostics & Advanced Technology, College of Animal Science and Technology & College of Veterinary Medicine, Zhejiang A&F University, Hangzhou, China

^3^Zhejiang International Science and Technology Cooperation Base for Veterinary Medicine and Health Management, College of Animal Science and Technology & College of Veterinary Medicine, Zhejiang A&F University, Hangzhou, China

^4^China-Australia Joint Laboratory for Animal Health Big Data Analytics, College of Animal Science and Technology & College of Veterinary Medicine, Zhejiang A&F University, Hangzhou, China

^5^Ningbo College of Health Sciences, Ningbo, China

*Correspondence: [lamge@zafu.edu.cn](mailto:sjz@cau.edu.cn); [songhh@zafu.edu.cn](mailto:songhh@zafu.edu.cn)

^#^Contributed equally

Running title: GshF suppresses *iiB^man^* to resist oxidative stress

Figures

**Figure S1** Deletion of *gshF* decreased the tolerance of *L. monocytogenes* to hydrogen peroxide

**Figure S2** Deletion of *gshF* decreased the bacterial proliferation efficiency and the virulence in mice

Tables

**Table S1** The primers used in this study

**Table S2** The transcription of genes differs between EGD-e and Δ*gshF* strains under stress conditions, or under stress conditions and normal conditions in EGD-e strain, or Δ*gshF* and EGD-e under normal conditions

**Figures S1**

**
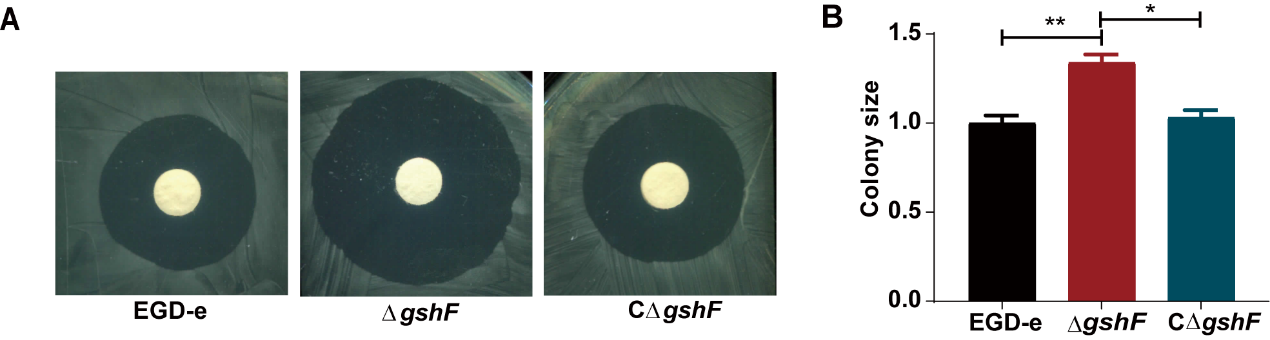
**

**Fig. S1 Deletion of *gshF* decreased the tolerance of *L. monocytogenes* to hydrogen peroxide**

(A) Overnight cultures of wild-type *L. monocytogenes* EGD-e, the *gshF* deletion strain Δ*gshF*, and the complement strain CΔ*gshF* were prepared. 0.1 mL portions of each culture were spread onto BHI agar plates. Subsequently, filter paper disks (diameter, 5 mm) were placed on the plates and soaked with 10 μL of 15% H_2_O_2_. The plates were then incubated overnight. The data presented in the study are based on three replicates. (B) The colony sizes of Δ*gshF* and CΔ*gshF* were compared to that of the EGD-e strain. Data are expressed as mean ± SEM of three replicates.*, P< 0.05; **, P< 0.01.

**Figures S2**


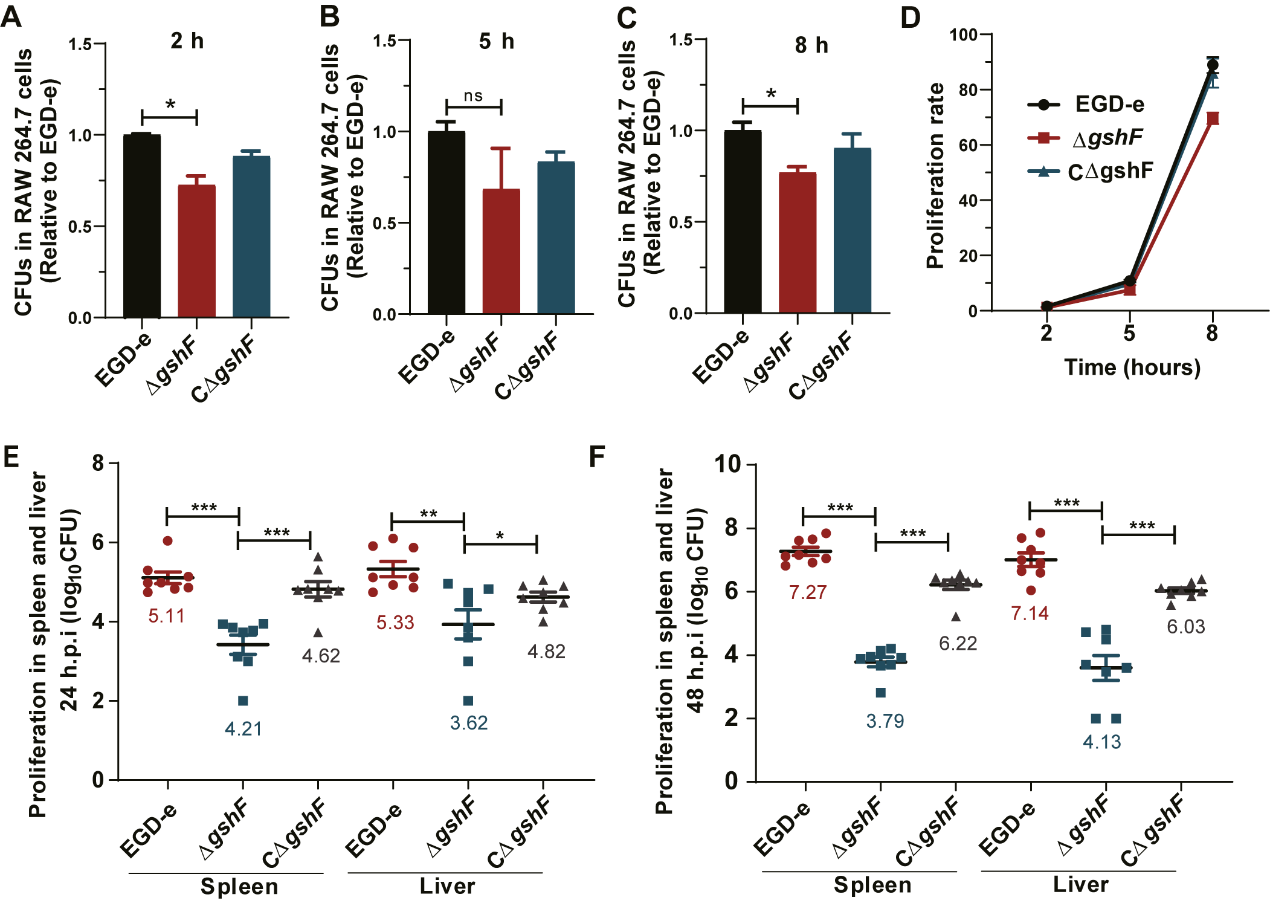


**Fig. S2** **Deletion of *gshF* decreased the bacterial proliferation efficiency and the virulence in mice**

1. D) The intracellular growth of wild-type *L. monocytogenes*, the *gshF* deletion strain Δ*gshF*, and the complement strain CΔ*gshF* was assessed in in RAW264.7 macrophages. The infected macrophages were lysed at 2 hours, 5 hours, and 8 hours post-infection, and viable bacteria were serially plated on BHI plates. The number of recovered bacteria was calculated as the mean ± SEM of three replicates for each strain (A-C). The proliferation rate was determined by dividing the total number of bacteria at each time point by the total number of bacteria invading cells at 1 h (D). Data are expressed as mean ± SEM of three replicates. (E, F) The proliferation of *L. monocytogenes* in mouse organs was examined. The wild-type and deletion strains were intraperitoneally inoculated into ICR mice at a concentration of approximately 4×10^6^ CFU. Animals were euthanized at 24 hours (E) or 48 hours (F) post-infection, and the organs (livers and spleens) were collected and homogenized. The homogenates were serially diluted and plated on BHI agar plates. The bacterial colonization in the organs is reported as the mean ± SEM of the log_10_ CFU per organ for each group, consisting of 8 mice. ns, no significance; *, P< 0.05; **, P< 0.01; ***, P< 0.001.

Table S1 The primers used in this study

| **primer** | **sequence（5’-3’）** | **plsmid** | **purpose** |
| --- | --- | --- | --- |
| Δ*gshF*-a-*BamH*I | CGCGGATCCCAACTGTCTGTACACTGTATGTATTAAAATGGTTCAT | pKSV7 | Delete *gshF* |
| Δ*gshF*-b | ATCTAAAATCTTATCTTCTAAACCAAAATGTCCAGAAAATAAAAGCTT |  |  |
| Δ*gshF*-c | CATTTTGGTTTAGAAGATAAGATTTTAGATTTCTTATTTGACTAAAAAAACCCA |  |  |
| Δ*gshF*-d-*Pst*I | TTGGTTCTGCAGGGCTGAAAATCCTTATTTAAAAACAAATGATTG |  |  |
| CΔ*gshF*-fwd-*Sac*I | TCCCCCGGGTTAGTCAAATAAGAAATCTAAAATCTTATCACCAATTTTTT | pIMK2 | Construct the complement strain of *gshF* deletion strain |
| CΔ*gshF*-rev-*Sma*I | CGAGCTCCCATCTTTTTAGTTAAGTTCCGAATTTTCATTATTC |  |  |
| Δ*lmo1997*-a-*Bam*HI | CGCGGATCCAGAAAAAGCGGCAGATAAATTTGGTCAAGAATT | pKSV7 | Delete *lmo1997* |
| Δ*lmo1997*-b | AAAATCTTCTTCTATAACAAAAATTGCTTTCATTTTCTAAACCGC |  |  |
| Δ*lmo1997*-c | AAAGCAATTTTTGTTATAGAAGAAGATTTTTAATAGGAAAAAGACAGC |  |  |
| Δ*lmo1997*-d-*Sal*I | ACGCGTCGACGGGGATAGTTTTTGGGCGATTTTATACATCG |  |  |
| Δ*lmo1997*-afront | TGATAAAATAACAAAATCCAAAATTTAATCAAAGAAAGTAGGAATG |  | Check the *lmo1997* deletion strain |
| Δ*lmo1998*-a-*Bam*HI | CGCGGATCCGCATACACAGGAATTGATGGAAGCGGAA | pKSV7 | Delete *lmo1998* |
| Δ*lmo1998*-b | ATAGTCTAACTGACGTTCATCAAATTTTAACACTCTTCATTCCTAC |  |  |
| Δ*lmo1998*-c | TTAAAATTTGATGAACGTCAGTTAGACTATTAAAAAGGCGGTTTAGA |  |  |
| Δ*lmo1998*-d-*Sal*I | ACGCGTCGACAGTTCGAGAGGGAAGATAAAAAAAGACAGCCG |  |  |
| Δ*lmo1998*-afront | GCAGTTATATATGTCGGATAAACTTGCTATTCCGGT |  | Check the *lmo1998* deletion strain |
| Δ*lmo1999*-a-*Bam*HI | CGCGGATCCTCTTCTGGGGAACTTTACGAGTTATCGCTACAG | pKSV7 | Delete *lmo1999* |
| Δ*lmo1999*-b | AATTTTGGATTTTGTATAATCCATGTTTTTCATTTTTTCACCTACTT |  |  |
| Δ*lmo1999*-c | AAAAACATGGATTATACAAAATCCAAAATTTAATCAAAGAAAGTAGGAA |  |  |
| Δ*lmo1999*-d-*Sal*I | ACGCGTCGACCTGCTAAATACATAAAACGATCTGCCACCATAAAGA |  |  |
| Δ*lmo1999*-afront | TTAGCTGTTTACTTAAAAGTTCCTGTAACAGGCGTAGC |  | Check the *lmo1999* deletion strain |
| Δ*lmo2000*-a-*Bam*HI | CGCGGATCCTGTTGTTAAAAATCTATGCTTTATTTTCGTATTACCTTACTTTGTT | pKSV7 | Delete *lmo2000* |
| Δ*lmo2000*-b | TTTTAATAAACCTGTAGAACTCTTCATCTTCATAATTTATACCTCCAT |  |  |
| Δ*lmo2000*-c | AAGATGAAGAGTTCTACAGGTTTATTAAAATAAGTAGGTGAAAAAATG |  |  |
| Δ*lmo2000*-d-*Sal*I | ACGCGTCGACTTCAACATGAGAATGGTTGCGCTATAGCC |  |  |
| Δ*lmo2000*-afront | CGAAGCAAAGAGAAGGAACAGCCAATTTTT |  | Check the *lmo2000* deletion strain |
| Δ*lmo2001*-a-*Bam*HI | CGCGGATCCATTTCTAAGAGTAGACCATCGTTTACTACATGGACAAGTAGC | pKSV7 | Delete *lmo2001* |
| Δ*lmo2001*-b | GAACTCTTCATCTTCTGTTCCAAACTGTTCCATAATTTATCCCTT |  |  |
| Δ*lmo2001*-c | GAACAGTTTGGAACAGAAGATGAAGAGTTCTGACGTATTAACGAA |  |  |
| Δ*lmo2001*-d-*Sal*I | ACGCGTCGACTGTTATAACCAATGAATAACCCATAATATCGAGTCAGAATAG |  |  |
| Δ*lmo2001*-afront | GGTCGTTCGCAAAAACGGTGTTGG |  | Check the *lmo2001*deletion strain |
| Δ*lmo2002*-a-*Bam*HI | CGCGGATCCAGAAATGACAGGTGTTATGAAAACTGATACCAATGAAA | pKSV7 | Delete *lmo2002* |
| Δ*lmo2002*-b | TGCTTCAAAGGTTACTCTTAGAAATTGAATCATTTTTTCTTCCT |  |  |
| Δ*lmo2002*-c | ATTCAATTTCTAAGAGTAACCTTTGAAGCATAAAAGACGAAAGAA |  |  |
| Δ*lmo2002*-d-*Sal*I | ACGCGTCGACTACCAATTGGCAAGTTGATCGATAAAAATCCA |  |  |
| Δ*lmo2002*-afront | ATGGGAGCTAAAAAGCCACTTTTTGAAGTAATTG |  | Check the *lmo2002* deletion strain |
| Δ*lmo2003*-a-*Bam*HI | CGCGGATCCCATGAGTAGTCAATTGCCGGGAGAACAAAA | pKSV7 | Delete *lmo2003* |
| Δ*lmo2003*-b | GTTAAAATCAACTACTGGCTTTTTAGCTCCCATTTCACTCATCC |  |  |
| Δ*lmo2003*-c | GGAGCTAAAAAGCCAGTAGTTGATTTTAACTAATACTAAGGAGGAAG |  |  |
| Δ*lmo2003*-d-*Sal*I | ACGCGTCGACCTTCAAAGGTTACTTTTTTTTCGTTAGGCACTTGC |  |  |
| Δ*lmo2003*-afront | TCACCAATCATCGGTGCGCAAAAAG |  | Check the *lmo2003* deletion strain |
| Δ*lmo2004*-a-*Bam*HI | GTGCCAAGCTTGCATGCCCATTACAACATGATATACCGGGAAG | pKSV7 | Delete *lmo2004* |
| Δ*lmo2004*-b | TTGATCGAATACTTTTTCTTGGATAAGCT |  |  |
| Δ*lmo2004*-c | GAAAAAGTATTCGATCAAAATGATGAACCGTTTGAGT |  |  |
| Δ*lmo2004*-d-*Sal*I | ATTACGAATTCGAGCTCGATAAACTCAACGGTAAAATTAAAC |  |  |
| Δ*lmo2004*-afront | GGTAACACTGGCATGGATGTTTCG |  | Check the *lmo2004* deletion strain |
| *lmo1997-lmo2004*+gfp-a-*Bam*HI | CGCGGATCCTCAAACAGAGCATGAGTTGGATTGGAAAATCT | pAM401 | Construct the P*_lmo2004_*-*gfp* fusion plasmid |
| *lmo1997-lmo2004*+gfp-b | CTTCTCCTTTACTCATATTTCCACCTCTTTTTTCAAATCATCTAT |  |  |
| *lmo1997-lmo2004*+gfp-c | AAAGAGGTGGAAATATGAGTAAAGGAGAAGAACTTTTCACTGGAG |  |  |
| *lmo1997-lmo2004*+gfp-d-*Sal*I | ACGCGTCGACTTATTTGTATAGTTCATCCATGCCATGTGTAATCCC |  |  |
| *lmo2770*-RT-fwd | TCTTCCGCTGGTAAAATGGG |  | Detect the expression of *lmo2770* |
| *lmo2770*-RT-rev | AAGACATACCACTCGCAGATG |  |  |
| *lmo2002* RT-fwd | TTGTCGAATCGGTTGAAGACGCTTA |  | Detect the expression of *lmo2002* |
| *lmo2002* RT-rev | TTCTCTTTGCTTCGTTCCGCCTAG |  |  |
| *lmo2004* RT-fwd | AGTCAATTGCCGGGAGAACA |  | Detect the expression of *lmo2004* |
| *lmo2004* RT-rev | ACACCGGCTTTTCTTCACTT |  |  |
| *rpoB* RT-rev | GGCTTCTTCCACTGTGCTCC |  | Detect the expression of *rpoB* |
| *rpoB* RT-fwd | CTACACTTAGGTATGGCTGCTCG |  |  |
| C*lmo1997*-*lmo2004_P_dlt_-*a | ACACCCGTCCTGTGGATCGTAATGTGCAAACCGTTATTTACC | pAM401 | Construct the *lmo1997*-*lmo2004* overexpression plasmid |
| C*lmo1997*-*lmo2004_P_dlt_-*b | ATATTCCATTATTGATATTCCCATTTTTAACAGTCC |  |  |
| C*lmo1997*-*lmo2004_P_dlt_-*c | ATATCAATAATGGAATATAAAGACTTGTCGCCAGC |  |  |
| C*lmo1997*-*lmo2004_P_dlt_-*d | CTCAAGGGCATCGGTCGATTAAAAATCTTCTTCTATTTCTTCAAGCGGC |  |  |
| C*lmo2002_P_dlt_-*a | ACACCCGTCCTGTGGATCCGTAATGTGCAAA | pAM401 | Construct the *lmo2002* overexpression plasmid |
| C*lmo2002_P_dlt_-*b | TTGAATCATTATTGATATTCCCATTTTTAACAGTCCTTTCT |  |  |
| C*lmo2002_P_dlt_-*c | ATATCAATAATGATTCAATTTCTAAGAGTAGACCATCGTTTACT |  |  |
| C*lmo2002_P_dlt_-*d | CTCAAGGGCATCGGTCGACTTATGCTTCAAAGGTTACTTTTTTTTCGTTAGGC |  |  |

Table S2 The transcription of genes differs between EGD-e and Δ*gshF* strains under stress conditions, or under stress conditions and normal conditions in EGD-e strain, or Δ*gshF* and EGD-e under normal conditions

| **gene** | **Oxidative stress of Cu^2+^** | **Oxidative stress of diamide** | **Oxidative stress of Cu^2+^** | **Normal conditions** | **gene name** | **Gene description** |
| --- | --- | --- | --- | --- | --- | --- |
|  | **log_2_fc（Δ*gshF*/EGD-e）** | **log_2_fc（Δ*gshF*/EGD-e）** | **log_2_fc（EGD-e stress/EGD-e non stress）** | **log_2_fc（Δ*gshF*/EGD-e）** |  |  |
| *lmo0004* | -1.98 | - | 4.14 | - | *lmo0004* | hypothetical protein |
| *lmo0005* | -2 | - | 4.57 | - | *recF* | recombination protein F |
| *lmo0006* | -1.22 | - | 1.21 | - | *gyrB* | DNA gyrase subunit B |
| *lmo0014* | 1.31 | - | - | - | *qoxB* | AA3-600 quinol oxidase subunit I |
| *lmo0015* | 1.78 | - | - | - | *qoxC* | AA3-600 quinol oxidase subunit III |
| *lmo0016* | 1.6 | - | - | - | *qoxD* | quinol oxidase aa3-600 subunit IV |
| *lmo0035* | 1.41 | - | - | - | *lmo0035* | glucosamine--fructose-6-phosphate aminotransferase |
| *lmo0076* | 1.11 | - | - | - | *lmo0076* | O6-methylguanine-DNA methyltransferase |
| *lmo0095* | -2.01 | - | 2.08 | - | *lmo0095* | hypothetical protein |
| *lmo0110* | 1.21 | - | -1.78 | - | *lmo0110* | lipase |
| *lmo0129* | 1.27 | - | - | - | *lmo0129* | N-acetylmuramoyl-L-alanine amidase |
| *lmo0133* | 1.16 | - | -2.31 | - | *lmo0133* | hypothetical protein |
| *lmo0134* | 1.06 | - | -1.46 | - | *lmo0134* | hypothetical protein |
| *lmo0135* | 1.59 | 1.62 | -1.40 | - | *lmo0135* | peptide ABC transporter substrate-binding protein |
| *lmo0136* | 1.36 | - | -1.71 | - | *lmo0136* | peptide ABC transporter permease |
| *lmo0140* | -1.32 | - | 1.25 | - | *lmo0140* | hypothetical protein |
| *lmo0149* | -1.03 | - | 2.04 | - | *lmo0149* | hypothetical protein |
| *lmo0154* | 2.34 | - | - | - | *lmo0154* | zinc ABC transporter ATP-binding protein |
| *lmo0155* | 1.33 | - | -1.80 | - | *lmo0155* | zinc ABC transporter permease |
| *lmo0169* | -1.09 | - | 2.64 | - | *lmo0169* | glucose transporter |
| *lmo0176* | -1.18 | - | 2.16 | - | *lmo0176* | glucose transporter |
| *lmo0177* | -1.29 | - | -1.59 | - | *metS* | methionyl-tRNA synthetase |
| *lmo0179* | 2.14 | - | -4.20 | - | *lmo0179* | sugar ABC transporter permease |
| *lmo0181* | 1.65 | - | -2.47 | - | *lmo0181* | sugar ABC transporter substrate-binding protein |
| *lmo0185* | -1.11 | - | 3.59 | - | *lmo0185* | hypothetical protein |
| *lmo0186* | -1.24 | 1.4 | 1.30 | - | *lmo0186* | hypothetical protein |
| *lmo0189* | -1.07 | - | 2.19 | - | *lmo0189* | Veg protein |
| *lmo0197* | 1.15 | - | - | - | *lmo0197* | regulatory protein SpoVG |
| *lmo0201* | 1.11 | - | -1.10 | -1.15 | *plcA* | phosphatidylinositol-specific phospholipase c |
| *lmo0202* | - | -1.42 | - | -1.17 | *hly* | cholesterol-dependent cytolysin listeriolysin O |
| *lmo0205* | -1.33 | - | - | - | *plcB* | phospholipase C |
| *lmo0211* | 1.47 | - | 2.50 | - | *ctc* | 50S ribosomal protein L25 |
| *lmo0217* | -1.39 | - | 3.22 | - | *lmo0217* | DivIC protein |
| *lmo0218* | -1.27 | - | 1.58 | - | *lmo0218* | hypothetical protein |
| *lmo0219* | -1.35 | - | 2.93 | - | *lmo0219* | hypothetical protein |
| *lmo0223* | 1.58 | 1.29 | - | - | *cysK* | cysteine synthase |
| *lmo0229* | 3.11 | - | 2.03 | - | *lmo0229* | CtsR family transcriptional regulator |
| *lmo0230* | 3.87 | - | 2.47 | - | *lmo0230* | hypothetical protein |
| *lmo0231* | 2.93 | - | 1.22 | - | *lmo0231* | ATP:guanido phosphotransferase |
| *lmo0232* | 1.95 | - | - | - | *clpC* | endopeptidase Clp ATP-binding chain C |
| *lmo0234* | -1.05 | - | 2.50 | - | *lmo0234* | hypothetical protein |
| *lmo0235* | -1.23 | - | 1.08 | - | *ispD* | 2-C-methyl-D-erythritol 4-phosphate cytidylyltransferase |
| *lmo0236* | -1.3 | - | 1.35 | - | *ispF* | 2-C-methyl-D-erythritol 2%2C4-cyclodiphosphate synthase |
| *lmo0239* | 2.12 | - | - | - | *cysS* | cysteinyl-tRNA synthetase |
| *lmo0240* | 1.63 | - | - | - | *lmo0240* | hypothetical protein |
| *lmo0246* | -1.5 | - | 2.11 | - | *nusG* | transcription antitermination protein NusG |
| *lmo0252* | -1.38 | - | 3.63 | - | *lmo0252* | penicilinase repressor |
| *lmo0253* | -1.05 | - | 2.86 | - | *lmo0253* | penicillinase antirepressor |
| *lmo0261* | 1.44 | - | - | - | *lmo0261* | phospho-beta-glucosidase |
| *lmo0264* | -1.83 | - | 1.27 | - | *inlE* | internalin E |
| *lmo0265* | 1.47 | - | - | - | *lmo0265* | succinyl-diaminopimelate desuccinylase |
| *lmo0267* | 1.18 | - | - | - | *lmo0267* | hypothetical protein |
| *lmo0270* | 1.06 | - | - | - | *lmo0270* | hypothetical protein |
| *lmo0272* | -2.06 | - | 3.17 | - | *lmo0272* | hypothetical protein |
| *lmo0274* | 1.07 | - | - | - | *lmo0274* | hypothetical protein |
| *lmo0276* | -1.15 | - | 2.98 | - | *lmo0276* | hypothetical protein |
| *lmo0278* | 1.85 | - | -3.64 | -1.34 | *lmo0278* | sugar ABC transporter ATP-binding protein |
| *lmo0281* | 1.51 | - | - | - | *lmo0281* | hypothetical protein |
| *lmo0292* | -1.2 | - | 1.47 | - | *lmo0292* | heat-shock protein htrA serine protease |
| *lmo0297** | 1.36 | - | -2.28 | - | *lmo0297* | transcriptional antiterminator BglG |
| *lmo0316* | 1.71 | - | -2.13 | - | *lmo0316* | hydroxyethylthiazole kinase |
| *lmo0317* | 1.48 | - | -2.78 | - | *lmo0317* | phosphomethylpyrimidine kinase |
| *lmo0318* | 1.34 | - | - | - | *thiE* | thiamine-phosphate pyrophosphorylase |
| *lmo0323* | 1.85 | - | -3.21 | - | *lmo0323* | hypothetical protein |
| *lmo0342* | 1.05 | -1.55 | -1.84 | - | *lmo0342* | transketolase |
| *lmo0343* | 1.22 | -1.61 | -2.13 | - | *lmo0343* | translaldolase |
| *lmo0344* | - | -1.82 | -1.68 | - | *lmo0344* | D-threitol dehydrogenase |
| *lmo0345* | - | -1.37 | -3.32 | - | *lmo0345* | ribose 5-phosphate isomerase B |
| *lmo0346* | - | -1.12 | -2.10 | - | *lmo0346* | triosephosphate isomerase |
| *lmo0347* | - | -1.07 | -2.68 | - | *lmo0347* | dihydroxyacetone kinase subunit L |
| *lmo0356* | 1.08 | - | - | - | *lmo0356* | oxidoreductase |
| *lmo0383* | 3.1 | - | -4.78 | - | *lmo0383* | methylmalonate-semialdehyde dehydrogenase |
| *lmo0386* | 1.12 | - | -2.57 | - | *lmo0386* | IolD protein |
| *lmo0392* | 1.06 | - | - | - | *lmo0392* | hypothetical protein |
| *lmo0398** | -3.27 | - | -3.27 | - | *lmo0398* | PTS sugar transporter subunit IIA |
| *lmo0403* | -1.25 | - | 2.57 | - | *lmo0403* | hypothetical protein |
| *lmo0419* | -1.07 | - | 1.31 | - | *lmo0419* | hypothetical protein |
| *lmo0425** | 1.55 | - | - | - | *lmo0425* | transcriptional antiterminator BglG |
| *lmo0426** | 1.75 | - | -2.63 | - | *lmo0426* | PTS fructose transporter subunit IIA |
| *lmo0427** | 1.65 | - | - | - | *lmo0427* | PTS fructose transporter subunit IIB |
| *lmo0428** | 1.95 | - | -2.82 | - | *lmo0428* | PTS fructose transporter subunit IIC |
| *lmo0429* | 1.18 | - | -2.25 | - | *lmo0429* | sugar hydrolase |
| *lmo0437* | 2.6 | - | - | - | *lmo0437* | hypothetical protein |
| *lmo0439* | 1.13 | - | - | - | *lmo0439* | hypothetical protein |
| *lmo0442* | -2.29 | - | - | - | *lmo0442* | hypothetical protein |
| *lmo0445* | 1.12 | - | 1.22 | - | *lmo0445* | transcripitonal regulator |
| *lmo0449* | -1.06 | - | - | - | *lmo0449* | hypothetical protein |
| *lmo0450* | -1.04 | - | - | - | *lmo0450* | hypothetical protein |
| *lmo0471* | -1.21 | - | - | - | *lmo0471* | hypothetical protein |
| *lmo0480* | 1.44 | - | - | - | *lmo0480* | transcriptional regulator |
| *lmo0481* | 1.13 | - | - | - | *lmo0481* | hypothetical protein |
| *lmo0482* | -1.13 | - | - | - | *lmo0482* | ribosomal RNA large subunit methyltransferase N |
| *lmo0489* | -1.09 | - | - | - | *lmo0489* | NADH:flavin oxidoreductase |
| *lmo0518* | 1.5 | - | -2.82 | - | *lmo0518* | hypothetical protein |
| *lmo0520* | 1.3 | - | -2.56 | - | *lmo0520* | transcriptional regulator |
| *lmo0521* | 1.74 | - | - | - | *lmo0521* | 6-phospho-beta-glucosidase |
| *lmo0546* | 2.49 | - | -3.41 | - | *lmo0546* | NAD(P)-dependent oxidoreductase |
| *lmo0547* | 1.09 | - | - | - | *lmo0547* | DeoR family transcriptional regulator |
| *lmo0553* | 1.24 | - | -1.54 | - | *lmo0553* | hypothetical protein |
| *lmo0579* | -1.04 | - | 3.04 | - | *lmo0579* | hypothetical protein |
| *lmo0582* | - | 1.69 | - | - | *lmo0582* | invasion associated endopeptidase |
| *lmo0604* | -3.2 | - | 4.27 | - | *lmo0604* | hypothetical protein |
| *lmo0606* | -1.32 | - | - | - | *lmo0606* | MarR family transcriptional regulator |
| *lmo0609* | 1.12 | - | 3.26 | - | *lmo0609* | phage shock protein E |
| *lmo0620* | -1.02 | - | 2.54 | - | *lmo0620* | hypothetical protein |
| *lmo0625* | 1.03 | - | -1.95 | - | *lmo0625* | hypothetical protein |
| *lmo0635* | -1.7 | - | 2.02 | - | *lmo0635* | hypothetical protein |
| *lmo0641* | 2.97 | - | -5.00 | - | *lmo0641* | heavy metal-transporting ATPase |
| *lmo0643* | 2.04 | - | -3.48 | - | *lmo0643* | transaldolase |
| *lmo0644* | -1.45 | - | 1.31 | - | *lmo0644* | hypothetical protein |
| *lmo0656* | -1.18 | - | 1.04 | - | *lmo0656* | hypothetical protein |
| *lmo0669* | 1.52 | - | - | - | *lmo0669* | oxidoreductase |
| *lmo0670* | 1.4 | - | - | - | *lmo0670* | hypothetical protein |
| *lmo0707* | -2.3 | - | - | - | *fliD* | flagellar capping protein FliD |
| *lmo0718* | -1.6 | - | - | - | *lmo0718* | hypothetical protein |
| *lmo0719* | 1.13 | - | -2.01 | - | *lmo0719* | hypothetical protein |
| *lmo0720* | 1.13 | - | -2.02 | - | *lmo0720* | hypothetical protein |
| *lmo0723* | -1.05 | - | - | - | *lmo0723* | metyl-accepting chemotaxis protein |
| *lmo0726* | 1.1 | - | - | - | *lmo0726* | hypothetical protein |
| *lmo0727* | 1.42 | - | - | - | *lmo0727* | glucosamine--fructose-6-phosphate aminotransferase |
| *lmo0729* | -2.56 | - | 5.05 | - | *lmo0729* | hypothetical protein |
| *lmo0730* | -1.7 | - | 5.07 | - | *lmo0730* | hypothetical protein |
| *lmo0735* | 2.13 | - | -4.80 | - | *lmo0735* | ribulose-5-phosphate 3-epimerase |
| *lmo0737* | 2.22 | - | -2.33 | - | *lmo0737* | hypothetical protein |
| *lmo0739* | - | -1.92 | - | - | *lmo0739* | glycoside hydrolase family 1 protein |
| *lmo0755* | -1.69 | - | -1.91 | - |  |  |
| *lmo0758* | 1.8 | - | 3.54 | - | *lmo0758* | hypothetical protein |
| *lmo0759* | 1.78 | - | 1.49 | - | *lmo0759* | hypothetical protein |
| *lmo0760* | 1.58 | - | - | - | *lmo0760* | hypothetical protein |
| *lmo0771* | -1.78 | - | 3.00 | - | *lmo0771* | hypothetical protein |
| *lmo0772* | -1.34 | - | 3.12 | - | *lmo0772* | transcriptional regulator |
| *lmo0778* | - | -1.49 | - | - | *lmo0778* | hypothetical protein |
| *lmo0789* | 2.07 | - | - | - | *lmo0789* | hypothetical protein |
| *lmo0790* | 1.81 | - | - | - | *lmo0790* | transcriptional regulator |
| *lmo0798* | -2.06 | - | -3.05 | - | *lmo0798* | lysine-specific permease |
| *lmo0800* | 1.59 | - | 1.35 | - | *lmo0800* | hypothetical protein |
| *lmo0810* | 1.14 | - | -2.03 | - | *lmo0810* | spermidine/putrescine ABC transporter substrate-binding protein |
| *lmo0811* | 1.81 | - | 1.78 | - | *lmo0811* | carbonic anhydrase |
| *lmo0814* | -1.92 | - | 1.54 | - | *lmo0814* | oxidoreductase |
| *lmo0815* | -1.62 | - | - | - | *lmo0815* | transcriptional regulator |
| *lmo0816* | -1.22 | - | 1.45 | - | *lmo0816* | regulatory protein PaiA |
| *lmo0822* | 2.23 | - | 2.08 | - | *lmo0822* | transcriptional regulator |
| *lmo0823* | 1.38 | - | 1.91 | - | *lmo0823* | oxidoreductase |
| *lmo0828* | 2.76 | - | -2.61 | - |  |  |
| *lmo0837* | -1.07 | - | -2.74 | - |  |  |
| *lmo0848* | 1.21 | - | -2.19 | - | *lmo0848* | amino acid ABC transporter ATP-binding protein |
| *lmo0851* | -1.22 | -1.19 | 4.77 | - | *lmo0851* | hypothetical protein |
| *lmo0855* | -1.23 | - | - | - | *ddl* | D-alanyl-alanine synthetase A |
| *lmo0866* | -1.63 | - | 1.18 | - | *lmo0866* | ATP-dependent RNA helicase |
| *lmo0867* | -1.77 | - | 3.86 | - | *lmo0867* | hypothetical protein |
| *lmo0871* | 2.03 | - | - | - | *lmo0871* | hypothetical protein |
| *lmo0880* | 3.21 | - | - | - | *lmo0880* | wall associated protein precursor |
| *lmo0881* | -2.23 | - | 4.63 | - | *lmo0881* | hypothetical protein |
| *lmo0884* | -1.11 | - | 2.07 | - | *lmo0884* | protoporphyrinogen oxidase |
| *lmo0903* | 2.39 | - | -2.64 | - | *lmo0903* | hypothetical protein |
| *lmo0906* | 1.13 | - | -1.61 | - | *lmo0906* | glutathione reductase |
| *lmo0907* | 1.14 | - | - | - | *lmo0907* | phosphoglycerate mutase |
| *lmo0919* | -1.31 | - | 2.90 | - | *lmo0919* | antibiotic ABC transporter ATP-binding protein |
| *lmo0920* | -1.24 | - | - | - | *lmo0920* | hypothetical protein |
| *lmo0930* | 1.32 | - | - | - | *lmo0930* | hypothetical protein |
| *lmo0944* | -1.02 | - | 2.16 | - | *lmo0944* | hypothetical protein |
| *lmo0945* | -1.13 | - | 2.21 | - | *lmo0945* | competence protein ComEC |
| *lmo0946* | -1.02 | - | 1.26 | - |  |  |
| *lmo0953* | 1.2 | - | 1.38 | - | *lmo0953* | hypothetical protein |
| *lmo0956* | 1.26 | - | - | - | *lmo0956* | N-acetylglucosamine-6P-phosphate deacetylase |
| *lmo0957* | 1.05 | - | - | - | *lmo0957* | glucosamine-6-phosphate isomerase |
| *lmo0958* | 1.06 | - | 1.94 | - | *lmo0958* | GntR family transcirptional regulator |
| *lmo0964^#^* | 1.37 | - | - | - | *lmo0964* | hypothetical protein |
| *lmo0971* | -1.23 | - | - | - | *dltD* | DltD protein for D-alanine esterification of lipoteichoic acid and wall teichoic acid |
| *lmo0972* | -1.7 | - | 1.94 | - | *dltC* | D-alanine--poly(phosphoribitol) ligase subunit 2 |
| *lmo0973* | -1.73 | - | 1.81 | - | *dltB* | DltB protein for D-alanine esterification of lipoteichoic acid and wall teichoic acid |
| *lmo0974* | -1.87 | - | 1.33 | - | *dltA* | D-alanine--poly(phosphoribitol) ligase subunit 1 |
| *lmo0983* | 1.54 | - | 1.02 | - | *lmo0983* | glutathione peroxidase |
| *lmo0984* | -1.56 | - | 3.52 | - | *lmo0984* | hypothetical protein |
| *lmo0986* | -1.1 | - | 1.65 | - | *lmo0986* | antibiotic ABC transporter ATP-binding protein |
| *lmo0987* | -1.23 | - | 1.77 | - | *lmo0987* | CylB protein |
| *lmo0988* | -1.28 | - | - | - | *prfC* | peptide chain release factor 3 |
| *lmo0989* | -1.06 | - | - | - | *lmo0989* | MarR family transcriptional regulator |
| *lmo0997* | 3.23 | - | 4.47 | - | *clpE* | ATP-dependent protease |
| *lmo1002** | - | 1.21 | - | - | *ptsH* | phosphocarrier protein HPr |
| *lmo1003** | - | 1.21 | - | - | *lmo1003* | phosphoenolpyruvate--protein phosphotransferase |
| *lmo1014* | -1.32 | - | 1.28 | - | *gbuA* | glycine/betaine ABC transporter ATP-binding protein |
| *lmo1017** | -1.24 | - | - | - | *lmo1017* | PTS glucose transporter subunit IIA |
| *lmo1018* | -1.31 | - | - | - | *lmo1018* | copper homeostasis protein CutC |
| *lmo1019* | -1.38 | - | 2.10 | - | *lmo1019* | hypothetical protein |
| *lmo1051* | 1.06 | - | 2.45 | - | *def* | peptide deformylase |
| *lmo1052* | 2.01 | - | 1.80 | - | *pdhA* | pyruvate dehydrogenase subunit E1 alpha |
| *lmo1053* | 1.98 | - | 1.29 | - | *PdhB* | pyruvate dehydrogenase subunit E1 beta |
| *lmo1054* | 1.7 | - | - | - | *pdhC* | dihydrolipoamide acetyltransferase |
| *lmo1055* | 1.78 | - | - | - | *PdhD* | dihydrolipoamide dehydrogenase |
| *lmo1057* | 1.09 | - | 3.65 | - | *lmo1057* | L-lactate dehydrogenase |
| *lmo1058* | 1.19 | - | 3.58 | - | *lmo1058* | hypothetical protein |
| *lmo1059^#^* | 1.7 | - | 1.44 | - | *lmo1059* | hypothetical protein |
| *lmo1067* | -1.71 | - | - | - | *lmo1067* | GTP-binding elongation factor |
| *lmo1069* | 1.2 | - | 11.205 | - | *lmo1069* | hypothetical protein |
| *lmo1076* | -1.23 | - | - | - | *lmo1076* | autolysin |
| *lmo1077* | -1.02 | - | - | - | *lmo1077* | teichoic acid biosynthesis protein B |
| *lmo1078* | -1.44 | - | 1.72 | - | *lmo1078* | UDP-glucose pyrophosphorylase |
| *lmo1079* | -1.04 | - | -1.65 | - | *lmo1079* | hypothetical protein |
| *lmo1080* | -1.2 | - | - | - | *lmo1080* | teichoic acid biosynthesis protein GgaB |
| *lmo1086* | -1.1 | - | 2.34 | - | *ispD* | 2-C-methyl-D-erythritol 4-phosphate cytidylyltransferase |
| *lmo1089* | -1.58 | 1.29 | - | - | *tagD* | glycerol-3-phosphate cytidylyltransferase |
| *lmo1091* | -1.14 | - | - | - | *lmo1091* | glysosyltransferase |
| *lmo1131* | -2.56 | - | 1.73 | - | *lmo1131* | ABC transporter ATP-binding protein |
| *lmo1132* | -1.76 | - | - | - | *lmo1132* | ABC transporter ATP-binding protein |
| *lmo1134* | -1.2 | - | 4.29 | - | *lmo1134* | regulatory protein |
| *lmo1135* | -1.15 | - | 3.59 | - | *lmo1135* | hypothetical protein |
| *lmo1137* | 1.82 | - | - | - | *lmo1137* | hypothetical protein |
| *lmo1138* | 2.79 | - | 2.00 | - | *lmo1138* | ATP-dependent Clp protease proteolytic subunit |
| *lmo1140* | 1.46 | - | - | - | *lmo1140* | hypothetical protein |
| *lmo1142* | 1.43 | - | -2.50 | - | *lmo1142* | PduS protein |
| *lmo1143* | 1.97 | - | -3.50 | - | *lmo1143* | PduT protein |
| *lmo1156* | 1.1 | - | - | - | *lmo1156* | diol dehydratase-reactivating factor large subunit |
| *lmo1189* | -1.02 | - | 1.27 | - | *lmo1189* | transcriptional regulator |
| *lmo1219* | 1.15 | - | - | - |  |  |
| *lmo1221* | - | 1.4 | 1.12 | - | *lmo1221* | phenylalanine--tRNA ligase subunit alpha |
| *lmo1225* | -1.44 | - | - | - | *lmo1225* | MarR family transcriptional regulator |
| *lmo1229* | -1.28 | - | 2.50 | - | *lmo1229* | hypothetical protein |
| *lmo1233^#^* | 1.71 | - | - | - | *trxA* | thioredoxin |
| *lmo1234* | 1.33 | - | - | - | *uvrC* | excinuclease ABC subunit C |
| *lmo1240* | -1.05 | - | - | - | *lmo1240* | hypothetical protein |
| *lmo1242* | 1.11 | - | - | - | *lmo1242* | hypothetical protein |
| *lmo1249* | -2.55 | - | 3.45 | - | *lmo1249* | hypothetical protein |
| *lmo1250* | -2.72 | - | 3.19 | - | *lmo1250* | antibiotic resistance protein |
| *lmo1254* | 1.5 | - | -4.01 | - | *lmo1254* | alpha%2Calpha-phosphotrehalase |
| *lmo1255** | 1.52 | - | -4.14 | - | *lmo1255* | PTS trehalose transporter subunit IIBC |
| *lmo1257* | -1.67 | - | - | - | *lmo1257* | hypothetical protein |
| *lmo1260* | -1.3 | - | 3.36 | - | *proB* | gamma-glutamyl kinase |
| *lmo1268* | 1.07 |  | - | - | *clpX* | ATP-dependent protease ATP-binding subunit ClpX |
| *lmo1269* | -1.06 | - | 2.11 | - | *lmo1269* | type I signal peptidase |
| *lmo1270* | -1.64 | - | - | - | *lmo1270* | type I signal peptidase |
| *lmo1271* | -1.51 | - | 2.10 | - | *lmo1271* | type I signal peptidase |
| *lmo1272* | -1.24 | - | 2.56 | - | *rbgA* | ribosomal biogenesis GTPase |
| *lmo1273* | -1.34 | - | 3.85 | - | *rnhB* | ribonuclease HII |
| *lmo1292* | -1.4 | - | 3.23 | - | *lmo1292* | glycerophosphodiester phosphodiesterase |
| *lmo1294* | -1.51 | - | 3.39 | - | *miaA* | tRNA delta(2)-isopentenylpyrophosphate transferase |
| *lmo1307* | - | 1.37 | - | - | *lmo1307* | DUF2785 domain-containing protein |
| *lmo1313* | -1.3 | - | 2.43 | - | *pyrH* | uridylate kinase |
| *lmo1315* | -1.72 | - | 3.06 | - | *lmo1315* | UDP pyrophosphate synthase |
| *lmo1316* | -1.25 | - | - | - | *cdsA* | phosphatidate cytidylyltransferase |
| *lmo1332* | 1.09 | - | -1.62 | - | *lmo1332* | GTPase EngC |
| *lmo1339* | - | 1.27 | - | - | *lmo1339* | ROK family glucokinase |
| *lmo1349* | 1.56 | - | -1.61 | - | *lmo1349* | glycine dehydrogenase subunit 1 |
| *lmo1350* | 1.51 | - | -1.52 | - | *lmo1350* | glycine dehydrogenase subunit 2 |
| *lmo1356* | -1.06 | - | - | - | *lmo1356* | acetyl-CoA carboxylase subunit (biotin carboxyl carrier subunit) |
| *lmo1364* | -1.43 | - | - | - | *cspL* | cold-shock protein |
| *lmo1367* | 1.88 | - | - | - | *lmo1367* | arginine repressor ArgR |
| *lmo1368* | 1.81 | - | 1.01 | - | *recN* | DNA repair protein |
| *lmo1369* | -1.52 | - | 1.64 | - | *lmo1369* | phosphotransbutyrylase |
| *lmo1379* | 1.15 | - | - | - | *lmo1379* | sporulation protein SpoJ |
| *lmo1380* | 1.19 | - | 2.15 | - | *lmo1380* | hypothetical protein |
| *lmo1381* | 2.62 | - | - | - | *lmo1381* | acylphosphatase |
| *lmo1384* | 1.78 | - | - | - | *lmo1384* | hypothetical protein |
| *lmo1387* | 1.86 | - | - | - | *lmo1387* | pyrroline-5-carboxylate reductase |
| *lmo1388* | 2.3 | - | -3.59 | - | *tcsA* | CD4+ T cell-stimulating antigen%2C lipoprotein |
| *lmo1396* | - | 1.51 | 1.90 | - | *lmo1396* | CDP-diacylglycerol--glycerol-3-phosphate 3-phosphatidyltransferase |
| *lmo1400* | -1.31 | - | - | - | *lmo1400* | N-acetyltransferase |
| *lmo1401* | -1.22 | - | 2.23 | - | *lmo1401* | hypothetical protein |
| *lmo1408* | 1.86 | - | - | - | *lmo1408* | hypothetical protein |
| *lmo1416* | -1.48 | - | - | - | *lmo1416* | hypothetical protein |
| *lmo1418* | -1.37 | - | -2.49 | - | *lmo1418* | hypothetical protein |
| *lmo1419* | -1.31 | - | - | - | *lmo1419* | hypothetical protein |
| *lmo1424* | -1.93 | - | 1.29 | - | *lmo1424* | manganese transporter |
| *lmo1431* | -1.24 | - | - | - | *lmo1431* | ABC transporter ATP-binding protein |
| *lmo1437* | -1.13 | - | 2.29 | - | *lmo1437* | aspartate-semialdehyde dehydrogenase |
| *lmo1438* | -1.66 | - | - | - | *lmo1438* | penicillin-binding protein |
| *lmo1445* | 1.41 | - |  | - | *zurR* | ZurR family transcriptional regulator |
| *lmo1446* | 1.08 | - | - | - | *zurM* | metal (zinc) transport protein (ABC transporter%2C permease) |
| *lmo1447* | 1.46 | - | - | - | *zurA* | metal (zinc) transport protein(ABC transporter%2C ATP-binding protein) |
| *lmo1449* | -1.4 | 1.31 | - | - | *lmo1449* | endonuclease IV |
| *lmo1450* | -1.66 | - | 2.62 | - | *lmo1450* | DEAD/DEAH box helicase |
| *lmo1459* | 1.13 | - | - | - | *glyQ* | glycyl-tRNA synthetase subunit alpha |
| *lmo1461* | -1.34 | - | -1.73 | - | *lmo1461* | hypothetical protein |
| *lmo1462* | -1.51 | - | - | - | *era* | GTP-binding protein Era |
| *lmo1472* | 1.6 | - | - | - | *dnaJ* | molecular chaperone DnaJ |
| *lmo1473* | 2.2 | - | 1.36 | - | *dnaK* | molecular chaperone DnaK |
| *lmo1474* | 2.1 | - | 3.05 | - | *grpE* | heat shock protein GrpE |
| *lmo1475* | 1.74 | - | 2.67 | - | *hrcA* | heat-inducible transcription repressor |
| *lmo1485* | -1.8 | - | 3.63 | - | *lmo1485* | hypothetical protein |
| *lmo1488* | -1.01 | - | 1.72 | - | *nadD* | nicotinic acid mononucleotide adenylyltransferase |
| *lmo1489* | -1.02 | - | 3.60 | - |  |  |
| *lmo1491* | -1.04 | - | 2.59 | - | *lmo1491* | GTP-binding protein |
| *lmo1492* | -1.11 | - | 3.08 | - | *lmo1492* | hypothetical protein |
| *lmo1496* | -1.02 | - | 2.05 | - | *greA* | transcription elongation factor GreA |
| *lmo1499* | -1.56 | - | - | - | *lmo1499* | hypothetical protein |
| *lmo1506* | -1.24 | - | 2.15 | - | *lmo1506* | transporter |
| *lmo1518* | -1.81 | - | 4.48 | - | *lmo1518* | hypothetical protein |
| *lmo1520* | 1.01 | - | - | - | *hisS* | histidyl-tRNA synthetase |
| *lmo1525* | -1.3 | - | -1.12 | - | *lmo1525* | recombination protein RecJ |
| *lmo1528* | -1.24 | - | - | - | *lmo1528* | hypothetical protein |
| *lmo1534* | 1.05 | - | - | - | *lmo1534* | L-lactate dehydrogenase |
| *lmo1536* | -1.17 | - | -1.12 | - | *lmo1536* | prephenate dehydratase |
| *lmo1537* | -1.69 | - | - | - | *obgE* | GTPase ObgE |
| *lmo1538* | 1.73 | - | -1.37 | - | *glpK* | glycerol kinase |
| *lmo1539* | 1.61 | - | -1.05 | - | *lmo1539* | glycerol transporter |
| *lmo1546* | - | 1.79 | 1.61 | - | *lmo1546* | rod shape-determining protein MreD |
| *lmo1547* | -1.35 | - | - | - | *mreC* | rod shape-determining protein MreC |
| *lmo1548* | -1.45 | - | 1.41 | - | *mreB* | rod shape-determining protein MreB |
| *lmo1551* | -1.52 | - | -1.61 | - | *folC* | folyl-polyglutamate synthetase |
| *lmo1558* | -1.34 | - | 2.19 | - | *engB* | GTP-binding protein EngB |
| *lmo1571* | - | 1.28 | - | - |  | 6-phosphofructokinase |
| *lmo1583* | 1.35 | - | - | - | *tpx* | thiol peroxidase |
| *lmo1586* | - | 1.23 | 2.32 | - |  | NAD kinase |
| *lmo1588* | 1.79 | - | -2.36 | - | *argD* | acetylornithine aminotransferase |
| *lmo1589* | 2.88 | - | - | - | *argB* | acetylglutamate kinase |
| *lmo1590* | 4.14 | - | -3.22 | - | *argJ* | bifunctional ornithine acetyltransferase/N-acetylglutamate synthase |
| *lmo1591* | 3.6 | - | -4.32 | - | *argC* | N-acetyl-gamma-glutamyl-phosphate reductase |
| *lmo1593* | -1.14 | - | - | - | *lmo1593* | iron-sulfur cofactor synthesis protein NifS |
| *lmo1594* | -1.33 | - | - | - | *lmo1594* | septation ring formation regulator EzrA |
| *lmo1596a* | -1.44 | - | 1.62 | - | *lmo1596a* | hypothetical protein |
| *lmo1597* | -1.75 | - | - | - | *lmo1597* | hypothetical protein |
| *lmo1600* | - | 1.32 | - | - | *lmo1600* | 3-deoxy-7-phosphoheptulonate synthase |
| *lmo1608* | 1.4 | - | - | - | *lmo1608* | hypothetical protein |
| *lmo1609^#^* | 1.86 | - | - | - | *lmo1609* | thioredoxin |
| *lmo1610* | - | 1.39 | 1.54 | - | *lmo1610* | conserved hypothetical protein |
| *lmo1617* | -1.61 | - | - | - | *lmo1617* | multidrug transporter |
| *lmo1618* | -1.59 | - | 2.01 | - | *lmo1618* | MarR family transcriptional regulator |
| *lmo1627* | 1.12 | - | - | - | *trpA* | tryptophan synthase subunit alpha |
| *lmo1628* | 1.71 | - | - | - | *trpB* | tryptophan synthase subunit beta |
| *lmo1629* | 1.56 | - | - | - | *trpF* | N-(5'-phosphoribosyl)anthranilate isomerase |
| *lmo1630* | 2.16 | - | - | - | *trpC* | indole-3-glycerol phosphate synthase |
| *lmo1631* | 1.72 | - | - | - | *trpD* | anthranilate phosphoribosyltransferase |
| *lmo1632* | 1.24 | - | - | - | *trpG* | anthranilate synthase subunit beta |
| *lmo1634* | -1.78 | - | - | - | *lmo1634* | bifunctional acetaldehyde-CoA/alcohol dehydrogenase |
| *lmo1636* | -1.37 | - | - | - | *lmo1636* | ABC transporter ATP-binding protein |
| *lmo1640* | 1.43 | - | - | - | *lmo1640* | hypothetical protein |
| *lmo1641* | 1.49 | - | -1.01 | - | *citB* | aconitate hydratase |
| *lmo1671* | 1.85 | - | -1.49 | - | *lmo1671* | ABC transporter |
| *lmo1674* | -1.06 | - | -1.92 | - | *lmo1674* | prolyl aminopetidase |
| *lmo1675* | -1.13 | - | - | - | *menD* | 2-succinyl-5-enolpyruvyl-6-hydroxy-3-cyclohexene-1-carboxylate synthase |
| *lmo1676* | -1.27 | - | - |  | *menF* | menaquinone-specific isochorismate synthase |
| *lmo1678* | 1.27 | - | - | - | *lmo1678* | bifunctional homocysteine S-methyltransferase/5%2C10-methylenetetrahydrofolate reductase |
| *lmo1679* | 1.31 | - | - | - | *lmo1679* | cystathionine beta-lyase |
| *lmo1682* | -1.01 | - | -1.23 | - |  |  |
| *lmo1690* | -1.69 | - | 3.12 | - | *lmo1690* | hypothetical protein |
| *lmo1692* | -1.2 | - | 2.23 | - | *lmo1692* | hypothetical protein |
| *lmo1704* | 1.71 | - | - | - | *lmo1704* | hypothetical protein |
| *lmo1705* | -1.52 | - | - | - | *lmo1705* | deoxyguanosine kinase/deoxyadenosine kinase |
| *lmo1716* | 2 | - | - | - | *lmo1716* | transcriptional regulator |
| *lmo1719** | -1.64 | - | - | - | *lmo1719* | PTS lichenan transporter subunit IIA |
| *lmo1720** | -2.29 | - | 2.06 | - | *lmo1720* | PTS lichenan transporter subunit IIB |
| *lmo1722* | -1.16 | - | - | - | *lmo1722* | ATP-dependent RNA helicase |
| *lmo1733* | 1.06 | - | - | - | *gltD* | glutamate synthase subunit beta |
| *lmo1739* | 1.2 | - | - | - | *lmo1739* | amino acid ABC transporter ATP-binding protein |
| *lmo1750* | -1.07 | - | - | - |  |  |
| *lmo1753* | -1.56 | - | 4.19 | - | *lmo1753* | lipid kinase |
| *lmo1756* | -1.04 | - | - | - | *gatC* | aspartyl/glutamyl-tRNA amidotransferase subunit C |
| *lmo1761* | -1.65 | - | -3.03 | - | *lmo1761* | sodium-dependent transporter |
| *lmo1777* | - | -1.2 | 3.73 | - | *lmo1777* | HD domain-containing protein |
| *lmo1786* | 2.09 | - | -2.74 | - | *inlC* | internalin C |
| *lmo1798* | 1.47 | - | - | - | *lmo1798* | hypothetical protein |
| *lmo1806* | -2.14 | - | -2.13 | - | *acpP* | acyl carrier protein |
| *lmo1817* | -1.13 | - | - | - | *lmo1817* | hypothetical protein |
| *lmo1830* | 1.37 | - | -1.19 | - | *lmo1830* | short-chain dehydrogenase |
| *Lmo1839* | - | - | - | -1.41 | *pyrP* |  |
| *lmo1840* | -1.99 | - | 3.84 | - | *pyrR* | bifunctional pyrimidine regulatory protein PyrR uracil phosphoribosyltransferase |
| *lmo1843* | -1.22 | - | - | - | *lmo1843* | hypothetical protein |
| *lmo1844* | -1.29 | - | - | - | *lspA* | lipoprotein signal peptidase |
| *lmo1847* | -1.49 | - | - | - | *lmo1847* | metal ABC transporter |
| *lmo1848* | -2.05 | - | - | - | *lmo1848* | metal ABC transporter permease |
| *lmo1849* | -2.02 | - | - | - | *lmo1849* | metal ABC transporter ATP-binding protein |
| *lmo1850* | -2.27 | - | 1.20 | - | *lmo1850* | MarR family transcriptional regulator |
| *lmo1851* | -2.4 | - | 4.17 | - | *lmo1851* | carboxy-terminal processing proteinase |
| *lmo1856* | -1.09 | - | - | - | *deoD* | purine nucleoside phosphorylase |
| *lmo1860^#^* | 1.04 | - | - | - | *lmo1860* | methionine sulfoxide reductase A |
| *lmo1864* | -1.58 | - | 5.55 | - | *lmo1864* | hemolysin |
| *lmo1866* | -1.08 | - | -1.05 | - | *lmo1866* | hypothetical protein |
| *lmo1869* | -1.36 | - | - | - | *lmo1869* | hypothetical protein |
| *lmo1871* | -1.21 | - | - | - | *lmo1871* | phosphoglucomutase |
| *lmo1875* | -1.04 | - | 2.43 | - | *lmo1875* | ABC transporter ATP-binding protein |
| *lmo1878* | -1.54 | - | 2.90 | - | *lmo1878* | manganese transport transcriptional regulator |
| *lmo1879* | -1.16 | - | - | - | *cspD* | cold-shock protein |
| *lmo1881* | 1.04 | - | - | - | *lmo1881* | 5'-3' exonuclease |
| *lmo1882* | 1.51 | - | 1.53 | - | *rpsN* | 30S ribosomal protein S14 |
| *lmo1883* | 1.06 | - | -1.01 | - | *lmo1883* | chitinase |
| *lmo1884* | -2.02 | - | - | - | *lmo1884* | xanthine permease |
| *lmo1885* | -1.71 | - | - | - | *lmo1885* | xanthine phosphoribosyltransferase |
| *lmo1886* | -1.15 | - | -1.55 | - | *lmo1886* | carboxypeptidase |
| *lmo1891* | -1.37 | - | 5.49 | - | *recU* | Holliday junction-specific endonuclease |
| *lmo1892* | -1.24 | - | - | - | *pbpA* | penicillin-binding protein 2A |
| *lmo1919* | -1.57 | - | 4.30 | - | *lmo1919* | hypothetical protein |
| *lmo1922* | -1.02 | - | 1.27 | - | *lmo1922* | hypothetical protein |
| *lmo1925* | -1.02 | - | -1.20 | - | *hisC* | histidinol-phosphate aminotransferase |
| *lmo1929* | -1.14 | - | -1.25 | - | *ndk* | nucleoside diphosphate kinase |
| *lmo1947* | -1.38 | - | - | - | *resE* | two-component sensor histidine kinas |
| *lmo1956* | -1.12 | - | 2.96 | - | *fur* | Fur family transcriptional regulator |
| *lmo1957* | -1.21 | - | - | - | *fhuG* | ferrichrome ABC transporter permease |
| *lmo1958* | -2.35 | - | - | - | *fhuB* | ferrichrome ABC transporter permease |
| *lmo1960* | -1.26 | - | 2.38 | - | *fhuC* | ferrichrome ABC transporter ATP-binding protein |
| *lmo1962* | 1.25 | - | - | - | *lmo1962* | TetR family transcriptional regulator |
| *lmo1963* | 1.89 | - | - | - | *lmo1963* | hypothetical protein |
| *lmo1964* | 1.77 | - | - | - | *lmo1964* | ABC transporter ATP-binding protein |
| *lmo1970* | 1.66 | - | -1.88 | - |  |  |
| *lmo1983* | -2.3 | - | - | - | *ilvD* | dihydroxy-acid dehydratase |
| *lmo1984* | -2.35 | - | - | - | *ilvB* | acetolactate synthase |
| *lmo1985* | -2.29 | - | 1.96 | - | *ilvH* | acetolactate synthase small subunit |
| *lmo1986* | -2.43 | -1.15 | - | - | *ilvC* | ketol-acid reductoisomerase |
| *lmo1987* | -2.37 | - | - | - | *leuA* | 2-isopropylmalate synthase |
| *lmo1993* | 1.06 | - | -6.08 | - | *pdp* | pyrimidine-nucleoside phosphorylase |
| *lmo1997** | 3.67 | 4.24 | - | - | *iiA^man^* | PTS mannose transporter subunit IIA |
| *lmo1998* | 4.08 | 4.19 | -3.01 | - | *lmo1998* | opine catabolism protein |
| *lmo1999* | 4.82 | 4.41 | -5.10 | - | *lmo1999* | hypothetical protein |
| *lmo2000** | 6.48 | 4.44 | -7.17 | - | *iiD^man^* | PTS mannose transporter subunit IID |
| *lmo2001** | 5.88 | 4.29 | -7.63 | - | *iiC^man^* | PTS mannose transporter subunit IIC |
| *lmo2002** | 6.08 | 4.13 | -7.85 | - | *iiB^man^* | PTS mannose transporter subunit IIB |
| *lmo2003* | 4.61 | 3.71 | -3.82 | - | *lmo2003* | GntR family transcriptional regulator |
| *lmo2004* | 4.14 | 4.9 | - | - | *lmo2004* | GntR family transcriptional regulator |
| *lmo2005* | 2.2 | - | - | - | *lmo2005* | oxidoreductase |
| *lmo2006* | 4.55 | - | - | - | *alsS* | acetolactate synthase |
| *lmo2007* | 1.83 | - | -1.90 | - | *lmo2007* | hypothetical protein |
| *lmo2013* | 1.81 | - | - | - | *lmo2013* | hypothetical protein |
| *lmo2014* | 1.4 | - | - | - | *lmo2014* | sugar hydrolase |
| *lmo2016* | 1.63 | - | -3.18 | - | *cspB* | cold-shock protein |
| *lmo2025* | 1.38 | - | - | - | *nadA* | quinolinate synthetase |
| *lmo2038* | -1.19 | - | -1.17 | - | *murE* | UDP-N-acetylmuramoylalanyl-D-glutamate--2%2C6-diaminopimelate ligase |
| *lmo2039* | -1.06 | - | - | - | *pbpB* | penicillin-binding protein 2B |
| *lmo2040* | -1.14 | - | - | - | *ftsL* | cell division protein FtsL |
| *lmo2049* | -1.11 | - | - | - | *lmo2049* | hypothetical protein |
| *lmo2051* | -1.45 | - | - | - | *lmo2051* | hypothetical protein |
| *lmo2052* | -1.12 | - | 1.24 | - | *coaD* | phosphopantetheine adenylyltransferase |
| *lmo2057* | 1.37 | - | 1.28 | - | *ctaB* | protoheme IX farnesyltransferase |
| *lmo2059* | -1.14 | - | - | - | *lmo2059* | potassium channel protein |
| *lmo2068* | 2.49 | - | - | - | *groEL* | molecular chaperone GroEL |
| *lmo2069* | 2.63 | - | 1.16 | - | *groES* | co-chaperonin GroES |
| *lmo2070* | -1 | - | 1.18 | - | *lmo2070* | hypothetical protein |
| *lmo2088* | -2.06 | - | 3.40 | - | *lmo2088* | transcriptional regulator |
| *lmo2090* | 4.17 | - | -1.40 | - | *argG* | argininosuccinate synthase |
| *lmo2091* | 3.3 | - | -1.64 | - | *argH* | argininosuccinate lyase |
| *lmo2094* | - | -1.17 | - | - | *lmo2094* | hypothetical protein |
| *lmo2104a* | -1.25 | - | 3.65 | - | *lmo2104a* | hypothetical protein |
| *lmo2105* | -1.49 | - | 2.25 | - | *lmo2105* | ferrous iron transport protein B |
| *lmo2114* | -2.24 | - | 3.03 | - | *lmo2114* | ABC transporter ATP-binding protein |
| *lmo2121* | - | -1.7 | -1.40 | - | *lmo2121* | glycoside hydrolase family 65 protein |
| *lmo2122* | - | -1.12 | -2.48 | - | *lmo2122* | conserved hypothetical protein |
| *lmo2132* | 1.56 | - | - | - | *lmo2132* | hypothetical protein |
| *lmo2149* | -1.71 | - | 3.18 | - | *lmo2149* | hypothetical protein |
| *lmo2151* | -1.32 | - | 2.44 | - | *lmo2151* | hypothetical protein |
| *lmo2152^#^* | 1.05 | - | - | - | *lmo2152* | thioredoxin |
| *lmo2156* | -2.69 | - | 3.14 | - | *lmo2156* | hypothetical protein |
| *lmo2162* | 1.73 | - | -2.79 | - | *lmo2162* | hypothetical protein |
| *lmo2163* | 1.64 | - | -1.47 | - | *lmo2163* | oxidoreductase |
| *lmo2171* | -1.89 | - | -3.53 | - | *lmo2171* | MFS transporter |
| *lmo2173* | -1.87 | - | - | - | *lmo2173* | sigma-54-dependent transcriptional regulator |
| *lmo2175* | 1.26 | - | -3.54 | - | *fabG* | 3-ketoacyl-ACP reductase |
| *lmo2177* | -1.3 | - | 2.03 | - | *lmo2177* | hypothetical protein |
| *lmo2185* | -1.75 | - | 1.70 | - | *lmo2185* | hypothetical protein |
| *lmo2195* | - | 1.43 | - | - | *lmo2195* | ABC transporter permease |
| *lmo2198* | - | 1.25 | -1.17 | - | *lmo2198* | tryptophan--tRNA ligase |
| *lmo2199* | 1.66 | - | - | - | *lmo2199* | hypothetical protein |
| *lmo2202* | -1.2 | - | -1.81 | - | *lmo2202* | 3-oxoacyl-ACP synthase |
| *lmo2205* | 1.08 | - | - | - | *lmo2205* | phosphoglyceromutase |
| *lmo2206* | 2.71 | - | 1.30 | - | *clpB* | Clp protease subunit B |
| *lmo2207* | -1.31 | - | 3.31 | - | *lmo2207* | hypothetical protein |
| *lmo2208* | -1.1 | - | 3.61 | - | *lmo2208* | hypothetical protein |
| *lmo2209* | -1.6 | - | 3.79 | - | *lmo2209* | hypothetical protein |
| *lmo2210* | -1.41 | - | 5.35 | - | *lmo2210* | hypothetical protein |
| *lmo2219* | -1.3 | - | 2.63 | - | *lmo2219* | foldase |
| *lmo2222* | -1.47 | - | 2.92 | - | *lmo2222* | hypothetical protein |
| *lmo2229* | -1.25 | - | - | - | *lmo2229* | penicillin-binding protein |
| *lmo2230* | 1.19 | - | - | - | *lmo2230* | arsenate reductase |
| *lmo2235* | -1.72 | - | - | - | *lmo2235* | NADH oxidase |
| *lmo2249* | 1.3 | - | -1.86 | - | *lmo2249* | low-affinity inorganic phosphate transporter |
| *lmo2250* | 1.69 | - | -1.38 | - | *arpJ* | amino acid ABC transporter permease |
| *lmo2251* | 1.59 | - | -1.92 | - | *lmo2251* | amino acid ABC transporter ATP-binding protein |
| *lmo2254* | - | -1.11 | -1.74 | - | *lmo2254* | NCS2 family permease |
| *lmo2256* | 1.3 | - | 1.32 | - | *lmo2256* | hypothetical protein |
| *lmo2274* | 1.27 | - | - | - | *lmo2274* | protein gp29 |
| *lmo2277* | 1.01 | - | - | - |  |  |
| *lmo2339* | 1.66 | - | -1.33 | - | *lmo2339* | hypothetical protein |
| *lmo2340* | 1.6 | - | -2.65 | - | *lmo2340* | hypothetical protein |
| *lmo2343* | 1.46 | - | - | - | *lmo2343* | nitrilotriacetate monooxygenase |
| *lmo2345* | 1.46 | - | - | - | *lmo2345* | hypothetical protein |
| *lmo2350* | 1.78 | - | -1.93 | - | *lmo2350* | hypothetical protein |
| *lmo2352* | 2.61 | - | - | - | *lmo2352* | LysR family transcriptional regulator |
| *lmo2360* | - | 1.72 | -2.35 | - | *lmo2360* | YhgE/Pip domain-containing protein |
| *lmo2373** | 1.3 | - | - | - | *lmo2373* | PTS beta-glucoside transporter subunit IIB |
| *lmo2390* | 1 | - | - | - |  |  |
| *lmo2391* | 1.44 | - | -1.27 | - | *lmo2391* | hypothetical protein |
| *lmo2392* | 2.21 | - | - | - | *lmo2392* | hypothetical protein |
| *lmo2393^#^* | 2.15 | - | - | - | *lmo2393* | hypothetical protein |
| *lmo2398* | 1.77 | - | -1.75 | - | *ltrC* | hypothetical protein |
| *lmo2406* | 1.59 | - | - | - | *lmo2406* | hypothetical protein |
| *lmo2407* | -1.3 | - | -1.71 | - | *lmo2407* | hypothetical protein |
| *lmo2410* | -1.72 | - | -2.82 | - | *lmo2410* | hypothetical protein |
| *lmo2425* | 1.57 | - | -1.56 | - | *lmo2425* | glycine cleavage system protein H |
| *lmo2426^#^* | 1.6 | - | 1.96 | - | *lmo2426* | hypothetical protein |
| *lmo2427* | -1.32 | - | -1.25 | - | *lmo2427* | cell division protein FtsW |
| *lmo2431* | -1.22 | - | - | - | *lmo2431* | ferrichrome ABC transporter substrate-binding protein |
| *lmo2432* | 2.86 | - | - | - | *lmo2432* | hypothetical protein |
| *lmo2434* | 1.22 | - | - | - | *lmo2434* | glutamate decarboxylase |
| *lmo2437* | 2.52 | - | - | - | *lmo2437* | hypothetical protein |
| *lmo2439* | -2.64 | - | 1.45 | - | *lmo2439* | hypothetical protein |
| *lmo2452* | 1.2 | - | 1.74 | - | *lmo2452* | carboxylesterase |
| *lmo2453* | 1.67 | - | - | - | *lmo2453* | epoxide hydrolase |
| *lmo2454* | 1.23 | - | -1.15 | - | *lmo2454* | hypothetical protein |
| *lmo2464* | - | -1.1 | 2.72 | - | *lmo2464* | TetR/AcrR family transcriptional regulator |
| *lmo2468* | 2.17 | - | 2.07 | - | *clpP* | ATP-dependent Clp protease proteolytic subunit |
| *lmo2469* | -1.02 | - | -1.96 | - | *lmo2469* | amino acid transporter |
| *lmo2478^#^* | 1.05 | - | - | - | *trxB* | thioredoxin reductase |
| *lmo2480* | -1.34 | - | 1.74 | - | *lmo2480* | acetyltransferase |
| *lmo2481* | -1.29 | - | - | - | *lmo2481* | pyrophosphatase PpaX |
| *lmo2482* | -1.52 | - | - | - | *lgt* | prolipoprotein diacylglyceryl transferase |
| *lmo2486* | -1.55 | - | 2.40 | - | *lmo2486* | hypothetical protein |
| *lmo2487* | -1.66 | - | 3.90 | - | *lmo2487* | hypothetical protein |
| *lmo2493* | -1.21 | - | 4.54 | - |  |  |
| *lmo2502* | -1.19 | - | - | - | *lmo2502* | hypothetical protein |
| *lmo2503* | -1.52 | - | - | - | *lmo2503* | cardiolipin synthase |
| *lmo2504* | -1.16 | - | -1.03 | - | *lmo2504* | cell wall-binding protein |
| *lmo2505* | -1.2 | - | - | - | *spl* | peptidoglycan lytic protein P45 |
| *lmo2507* | -1.06 | - | 4.18 | - | *ftsE* | cell division protein FtsE |
| *lmo2511* | 1.36 | - | -1.04 | - | *lmo2511* | hypothetical protein |
| *lmo2519* | -1.32 | - | - | - | *lmo2519* | teichoic acid linkage unit synthesis protein |
| *lmo2520* | -1.19 | - | - | - | *lmo2520* | O-succinylbenzoate-CoA synthase |
| *lmo2522* | - | 2.55 | 1.67 | - | *lmo2522* | LysM peptidoglycan-binding domain-containing protein |
| *lmo2536a* | -1.13 | - | - | - | *lmo2536a* | hypothetical protein |
| *lmo2537* | -1.04 | - | - | - | *lmo2537* | UDP-N-acetylglucosamine 2-epimerase |
| *lmo2539* | 1.67 | - | - | - | *glyA* | serine hydroxymethyltransferase |
| *lmo2547* | -1.08 | - | - | - | *hom* | homoserine dehydrogenase |
| *lmo2550* | -1.24 | - | -2.08 | - | *lmo2550* | glycosyl transferase |
| *lmo2551* | -1.35 | - | - | - | *rho* | transcription termination factor Rho |
| *lmo2553* | -1.19 | - | 1.54 | - | *lmo2553* | hypothetical protein |
| *lmo2554* | -1.05 | - | - | - | *lmo2554* | galactosyltransferase |
| *lmo2555* | -1.03 | - | 1.79 | - | *lmo2555* | N-acetylglucosaminyl-phosphatidylinositol biosynthesis protein |
| *lmo2562* | -1.16 | - | - | - | *lmo2562* | hypothetical protein |
| *lmo2564* | 1.41 | - | -1.80 | - | *lmo2564* | 4-oxalocrotonate isomerase |
| *lmo2567* | -3.25 | - | 7.14 | - | *lmo2567* | hypothetical protein |
| *lmo2568* | -3.92 | - | 4.84 | - | *lmo2568* | hypothetical protein |
| *lmo2569* | 2.26 | - | - | - | *lmo2569* | peptide ABC transporter substrate-binding protein |
| *lmo2574* | 2.57 | - | -1.73 | - | *lmo2574* | hypothetical protein |
| *lmo2575* | 2.6 | - | 2.63 | - | *lmo2575* | cation transporter |
| *lmo2585* | 2.02 | -1.03 | -5.49 | - | *lmo2585* | hypothetical protein |
| *lmo2586* | 1.64 | - | -6.31 | - | *lmo2586* | formate dehydrogenase subunit alpha |
| *lmo2587* | - | 1.4 | - | - | *lmo2587* | DNA-directed RNA polymerase subunit delta |
| *lmo2592* | 1.37 | - | -2.18 | - | *lmo2592* | aldo/keto reductase |
| *lmo2593* | 1.36 | - | -2.09 | - | *lmo2593* | MerR family transcriptional regulator |
| *lmo2596* | - | 1.47 | - | - | *lmo2596* | 30S ribosomal protein S9 |
| *lmo2597* | -1.22 | - | -1.44 | - | *rplM* | 50S ribosomal protein L13 |
| *lmo2606* | 1.01 | - | - | - | *rpoA* | DNA-directed RNA polymerase subunit alpha |
| *lmo2634* | -1.1 | - | - | - | *lmo2634* | hypothetical protein |
| *lmo2635* | -1.15 | - | 2.42 | - | *lmo2635* | 1%2C4-dihydroxy-2-naphthoate octaprenyltransferase |
| *lmo2636* | -1.37 | - | - | - | *lmo2636* | hypothetical protein |
| *lmo2642* | -1.51 | - | - | - | *lmo2642* | hypothetical protein |
| *lmo2644* | -1.09 | -1.18 | 3.02 | - | *lmo2644* | hypothetical protein |
| *lmo2651** | -2.43 | - | -5.06 | - | *lmo2651* | PTS mannitol transporter subunit IIA |
| *lmo2670* | 1.07 | - | - | - | *lmo2670* | hypothetical protein |
| *lmo2683** | -3.42 | - | -1.46 | - | *lmo2683* | PTS cellbiose transporter subunit IIB |
| *lmo2684** | -1.93 | - | -3.90 | - | *lmo2684* | PTS cellbiose transporter subunit IIC |
| *lmo2685** | -1.13 | - | -4.83 | - | *lmo2685* | PTS cellbiose transporter subunit IIA |
| *lmo2686* | -1.32 | - | - | - | *lmo2686* | hypothetical protein |
| *lmo2687* | 1.19 | - | -2.15 | - | *lmo2687* | cell division protein FtsW |
| *lmo2692* | 1.14 | - | 1.10 | - | *lmo2692* | hypothetical protein |
| *lmo2695* | 1.38 | - | - | - | *lmo2695* | dihydroxyacetone kinase subunit DhaK |
| *lmo2696* | 1.11 | - | - | - | *lmo2696* | dihydroxyacetone kinase |
| *lmo2697** | 1.02 | - | - | - | *lmo2697* | PTS mannose transporter subunit IIA |
| *lmo2705* | 1.39 | - | 1.85 | - | *lmo2705* | hypothetical protein |
| *lmo2707* | 1.48 | - | 1.46 | - | *lmo2707* | hypothetical protein |
| *lmo2708** | -2.68 | - | -7.10 | - | *lmo2708* | PTS cellbiose transporter subunit IIC |
| *lmo2709* | -1.7 | - | - | - | *lmo2709* | hypothetical protein |
| *lmo2710* | -1.45 | - | 1.72 | - | *lmo2710* | hypothetical protein |
| *lmo2714* | -1.19 | - | 1.12 | - | *lmo2714* | pepdidoglycan bound protein |
| *lmo2724* | 1.68 | - | - | - | *lmo2724* | hypothetical protein |
| *lmo2726* | -2.6 | - | - | - | *lmo2726* | MarR family transcriptional regulator |
| *lmo2727* | -1.16 | - | 1.93 | - | *lmo2727* | hypothetical protein |
| *lmo2729* | 1.43 | - | 2.12 | - | *lmo2729* | hypothetical protein |
| *lmo2730* | 1.37 | - | - | - | *lmo2730* | phosphatase |
| *lmo2731* | 1.36 | - | - | - | *lmo2731* | RpiR family transcriptional regulator |
| *lmo2733** | 1.13 | - | -1.24 | - | *lmo2733* | PTS fructose transporter subunit IIABC |
| *lmo2739* | 1.3 | - | 1.29 | - | *lmo2739* | NAD-dependent deacetylase |
| *lmo2742* | 2.75 | - | -1.30 | - | *lmo2742* | hypothetical protein |
| *lmo2743* | 2.17 | - | -1.99 | - | *lmo2743* | translaldolase |
| *lmo2746* | 1.52 | - | - | - | *lmo2746* | hypothetical protein |
| *lmo2747* | 1.13 | - | -2.44 | - | *serS* | seryl-tRNA synthetase |
| *lmo2759* | 2.4 | - | - | - | *lmo2759* | hypothetical protein |
| *lmo2764* | 1.3 | - | -3.62 | - | *lmo2764* | ROK family transcriptional regulator |
| *lmo2767* | -1.33 | - | 2.16 | - | *lmo2767* | hypothetical protein |
| *lmo2768* | -1.17 | - | - | - | *lmo2768* | hypothetical protein |
| *lmo2770* | -16.19 | -9.49 | - | - | *lmo2770* | bifunctional glutamate--cysteine ligase/glutathione synthetase |
| *lmo2779* | -1.31 | - | 1.40 | - | *lmo2779* | GTP-binding protein EngD |
| *lmo2792* | 1.48 | - | - | - | *lmo2792* | hypothetical protein |
| *lmo2814* | -1.18 | - | - | - | *lmo2814* | TetR family transcriptional regulator |
| *lmo2824* | 1.31 | - | - | - | *lmo2824* | D-3-phosphoglycerate dehydrogenase |
| *lmo2828* | 1.73 | - | - | - | *lmo2828* | hypothetical protein |
| *lmo2830^#^* | 2.44 | - | - | - | *lmo2830* | thioredoxin |
| *lmo2844* | -1.64 | - | 2.25 | - | *lmo2844* | hypothetical protein |
| *lmo2852* | -1.64 | - | -2.15 | - | *lmo2852* | hypothetical protein |

-: The genes listed in the table did not show significant differences in transcript levels.

*^*^* : The genes involved in phosphoenolpyruvate-carbohydrate phosphotransferase system (PTS)

*^#^*: The genes involved in thiol: disulfide redox metabolism

Log_2_ fc < -1 indicates the gene transcript level is downregulated, log_2_ fc > 1 indicates the gene transcript level is upregulated, and the genes listed in the table exhibit significant differences in transcript level
